# Contamination of herbal medicinal products in low-and-middle-income countries: A systematic review

**DOI:** 10.1016/j.heliyon.2023.e19370

**Published:** 2023-08-25

**Authors:** Kwabena F.M. Opuni, James-Paul Kretchy, Kofi Agyabeng, Joseph A. Boadu, Theodosia Adanu, Samuel Ankamah, Alexander Appiah, Geralda B. Amoah, Mariam Baidoo, Irene A. Kretchy

**Affiliations:** a Department of Pharmaceutical Chemistry, School of Pharmacy, University of Ghana, P.O. Box LG43, Legon, Accra, Ghana; b Department of Public Health, School of Medicine and Health Sciences, Central University, P. O. Box 2305, Miotso, Accra, Ghana; c Department of Biostatistics, School of Public Health, University of Ghana, P. O. Box LG13, Legon, Accra, Ghana; d Balme Library, University of Ghana, P.O. Box LG24, Legon, Accra, Ghana; e Department of Pharmacy Practice and Clinical Pharmacy, School of Pharmacy, University of Ghana, P.O. Box LG43, Legon, Accra, Ghana

**Keywords:** Microbial contamination, Chemical contamination, Metals, Pesticide residues, Mycotoxins, Residual solvents, Herbal medicinal products, Low-middle-income countries

## Abstract

The use of herbal medicinal products (HMPs) has grown significantly across low-and-middle-income countries (LMICs). Consequently, the safety of these products due to contamination is a significant public health concern. This systematic review aimed to determine the prevalence, types, and levels of contaminants in HMPs from LMICs. A search was performed in seven online databases, i.e., Africa journal online (AJOL), Cumulative Index to Nursing and Allied Health Literature (CINAHL), Directory of Open Access Journals (DOAJ), Health Inter-Network Access to Research Initiative (HINARI), World Health Organization Global Index Medicus (WHO GIM), Scopus, and PubMed using appropriate search queries and reported as per the “Preferred Reporting Items for Systematic Reviews and Meta-Analyses” (PRISMA) guidelines. Ninety-one peer-reviewed articles published from 1982 to 2021 from 28 different countries across four continents were included in the study. Although metals, microbial, mycotoxins, pesticides, and residual solvents were the reported contaminants in the 91 articles, metals (56.0%, 51/91), microbial (27.5%, 25/91), and mycotoxins (18.7%, 17/91) were the most predominant. About 16.4% (1236/7518) of the samples had their contaminant levels above the regulatory limits. Samples tested for microbial contaminants had the highest proportion (46.4%, 482/1039) of contaminants exceeding the regulatory limit, followed by mycotoxins (25.8%, 109/423) and metals (14.3%, 591/4128). The proportion of samples that had their average non-essential metal contaminant levels above the regulatory limit was (57.6%, 377/655), 18.3% (88/480), 10.7% (24/225), and 11.3% (29/257) for Pb, Cd, Hg, and As, respectively. The commonest bacteria species found were *Escherichia coli* (52.3%, 10/19) and *Salmonella species* (42.1%, 8/19). This review reported that almost 90% of *Candida albicans* and more than 80% of moulds exceeded the required regulatory limits. HMP consumption poses profound health implications to consumers and patients. Therefore, designing and/or implementing policies that effectively regulate HMPs to minimize the health hazards related to their consumption while improving the quality of life of persons living in LMICs are urgently needed.

## Highlights

• A systematic review of contaminants in HMPs using PRISMA guidelines was conducted.
• The study included 91 articles from 1982 to 2021 in 28 countries across 4 continents.
• Metals, microbial, and mycotoxins were the most predominant contaminants identified.
• Almost 16% of the samples had their contaminant levels above the regulatory limits.
• The highest proportion of contaminants exceeding the regulatory limit was microbial.

## 1 Introduction

The use of HMPs is central to many traditional, complementary, and alternative medicine practices worldwide. It is estimated that up to four billion people living in the developing world, comprising LMICs, rely on herbal medicine as a primary source of healthcare [[1], [2], [3], [4]]. A high prevalence of HMPs for treating and managing diseases has been reported in some LMICs [[5], [6], [7], [8], [9], [10]]. The consumption of HMPs globally has increased in recent decades, partly due to the widespread assumption that ‘natural’ implies ‘harmless’, which may not be entirely true [11]. Also, consumers and patients usually perceive HMPs as organic, less toxic, and safe since they are naturally sourced [12,13].

Although HMPs are widely accepted and used in LMICs [14], there is a significant concern for the safety of these products [11] due to contamination [15]. For example, HMPs in LMICs have been reported to contain microbiological [11] and chemical contaminants such as pesticide residues, residual solvents, mycotoxins, and heavy metals [[15], [16], [17]]. Poor handling and storage of HMPs may result in microbial and mycotoxin contamination [11,[18], [19], [20], [21]], and using organic solvents to manufacture these products may lead to residual solvent contamination [22]. The indiscriminate use of pesticides for farming purposes in LMICs has also resulted in the contamination of HMPs [[23], [24], [25]]. Also, anthropogenic activities have been implicated in heavy metal contamination of HMPs [[26], [27], [28], [29]]. These contaminants in HMPs have adverse health implications for consumers and patients, especially in LMICs where health systems are already challenged [30]. Exposure to contaminated HMPs from biological and chemical origins could lead to adverse health consequences and public health threats to these consumers and patients [31]. Elemental impurities in HMPs may cause liver and kidney problems, gastrointestinal disorders, hyperthyroidism, psychological and neurological disorders, nervous system abnormalities, cancers, and lung damage [[32], [33], [34]]. Microbial contamination of HMPs could also negatively affect consumers and patients because of immunocompromised conditions and microbial infections [35]. Mycotoxins may cause liver cancer, convulsions, and respiratory problems, reduce immunity, and alter protein metabolism in humans [36]. Also, pesticide exposure and consumption may cause cancer, neurological effects, diabetes, respiratory diseases, fetal diseases, and genetic disorders in humans [[37], [38], [39], [40]]. Residual solvents are carcinogenic, environmentally hazardous, neurotoxic, and teratogenic [41].

The adverse health implications associated with consuming contaminated HMPs in LMICs are further compounded by weak regulatory frameworks for manufacturing and distributing HMPs [42]. Knowledge of contaminants will help identify regulatory challenges and draw lessons for addressing the safety of HMPs.

This study aimed to determine the prevalence, types, and levels of contaminants found in the HMPs in LMICs. Relative to previous reviews [15,[43], [44], [45], [46], [47], [48], [49], [50]], this study sought to assess all possible known contaminants comprehensively. Again, unlike some previously reported reviews, a systematic review approach using the PRISMA reporting guidelines was adopted [15,[43], [44], [45], [46], [47], [48], [49], [50]]. The outcome of this systematic review would help in policy decision-making, enhance effective regulation of HMPs, and minimize possible health problems related to HMP use among persons living in LMICs.

## 2 Methodology

This systematic review was conducted and reported according to the Preferred Reporting Items for Systematic Reviews and Meta-Analyses (PRISMA) guideline [51]. The protocol for this study was registered in the Prospective Register of Systematic Reviews (PROSPERO, reference number: CRD42021229536) on 5^th^ February 2021.

### 2.1 Eligibility criteria

This study's inclusion and exclusion criteria were predetermined and form part of the study protocol. Primary laboratory-based quantitative studies were included if they (i) reported numerical contamination levels of HMPs, (ii) were conducted in LMICs, (iii) indicated relevant information for data analysis, and (iv) were published in English. HMPs included in this review were (i) herbal preparations (formulated product) of one or more herbs and/or excipients and (ii) classified as either a herb or spice, which was based on WHO's definition of HMPs [52].

A study was excluded if it: (i) was secondary (such as meta-analysis and review articles), (ii) did not report empirical data, c) was not available (full text) for analysis, (iv) has been retracted, (v) focused on method development using fortification of samples with contaminants instead of application samples, and (vi) was from high-income country contexts.

### 2.2 Search strategy

The search terminologies such as LMICs, HMPs, and contamination derived from the review questions and their synonyms were combined to create search queries using the Boolean operators and revised as required for maximum results (Table S1).

The concepts identified in the review question using the PICO (population; indicator; comparison; outcome) [53] are P - LMICs as recognized by World Bank [54], I - contamination of HMPs, C - not applicable, and O - any chemical contaminants with suspected or known connections to health.

The following online databases were searched without restriction: Africa journal online (AJOL), Cumulative Index to Nursing and Allied Health Literature (CINAHL), Directory of Open Access Journals (DOAJ), Health Inter-Network Access to Research Initiative (HINARI), World Health Organization Global Index Medicus (WHO GIM), Scopus, and PubMed. The online databases were searched between 14th February to 19^th^ April 2021 by TA and SA. Hand searches were done using Google Scholar on 11^th^ November 2021 to obtain additional information by KFMO.

### 2.3 Data extraction

The output from the database and hand searches were imported into the EndNote version X9 library [55]. Duplicate citations were removed using EndNote version X9 library based on consensus among team members. The resulting EndNote file was uploaded to the review management software, Rayyan, for screening [56]. Titles and abstracts were initially screened by AA, GBA, MB, JAB, and KFMO for eligibility. The full-text screening was performed independently for all potentially relevant studies by AA, GBA, MB, JAB, and KFMO. Any discrepancies at any stage were resolved with other team members.

The extracted data captured information on (i) full citation with a year of publication, (ii) country study was conducted, (iii) HMP analyzed (name of product, dosage/formulation form, active components of the preparation (where applicable), source of samples (including location), (iv) analysis of HMP samples (i.e., assay method used, the total number of samples analyzed), and (v) analysis for contaminants (i.e., type of contaminant, the subtype of contaminant, name, and number of contaminants tested, levels of contaminants detected). The extracted data on each sample was used in this study (sample level).

### 2.4 Quality appraisal

An analytical tool adapted from previous systematic reviews on herbal medicines was used to assess and evaluate the quality of the included articles [57,58]. The analytical method, contaminant characteristics, country of study, HMP analyzed, and health outcomes were appraised using the analytical tool. Table S2 shows the quality appraisal scoring system with a 1 point awarded to each quality appraisal dimension assessed and a possible maximum score of 14. Articles with summated scores ranging from 10 to 14, 6 to 9, and 0 to 5 were considered good, fair, and poor quality, respectively.

### 2.5 Data analysis

After being imported from Microsoft Excel, the data were analyzed at the article and sample levels with STATA version 15 (StataCorp LLC, USA). A world map was used to show the geographical distribution of the articles. The continental origin of the papers was assigned to the articles based on the country where the study was conducted. The distribution of the characteristics of the reviewed articles was analyzed using the articles as the unit of analysis. Thus, the data were collapsed from the sample to the article level before the analysis. Doughnut plots and bar charts were used to show the distribution of the study findings and characteristics. The contaminant concentration status of the samples was categorized into “0 – Below Limit” or “1 – Above Limit” by comparing the reported mean concentrations of the sample with the regulatory limit for the specific contaminant under consideration. The regulatory limits for the metals were obtained from either the general chapter <232> *Elemental Impurities – Limits* of United States Pharmacopoeia (USP) [59] or the European Medicines Agency (EMA) guideline on the specification limits for residues of metal catalysts or metal reagents [60]. The microbial contamination limits were obtained from the WHO guidelines for assessing the quality of herbal medicines [61]. The regulatory limits for the mycotoxins (aflatoxins, deoxynivalenol, fumonisins, ochratoxins, patulin, and zearalenone) were obtained from various guidelines such as USP [62], United States Food and Drugs Authority (USFDA) [63], European Food Safety Authority (EFSA) [64], EFSA [65], USFDA [66], and World Food Programme (WFP) [67]. Regulatory limits for the pesticides were obtained from WHO guidelines [61] except for ethoprophos, bifenthrin, and cyhalothrin, which were obtained from Codex [68]. Residual solvents regulatory limits were obtained from the general chapter <467> *Residual Solvents* in the USP [69]. In cases where the mean concentration was reported as either “undetected”, “acceptable”, or “not detected”, they were classified as “0 – Below Limit” while those reported as “not acceptable” were classified as “1 – Above Limit”. To show contaminants that predominantly exceeded the regulatory limits, a contingency table was used to indicate the contaminant concentration status by contaminant type. The unit of analysis for the contaminant concentration status was at the sample level.

## 3 Study characteristics

**Fig. 1:**
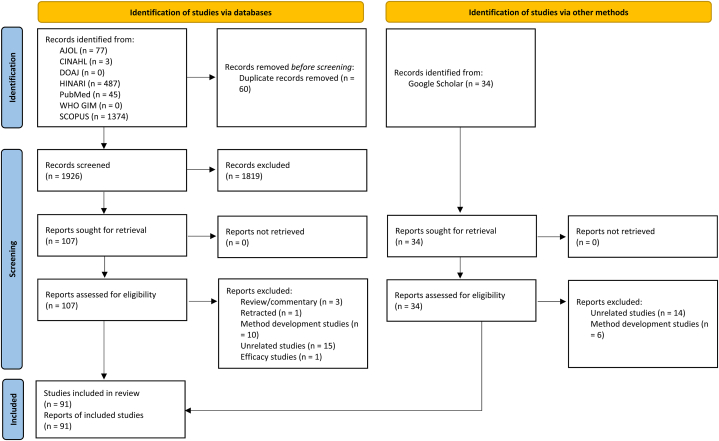
PRISMA flow chart for the systematic review. AJOL, Africa journal online; CINAHL, Cumulative Index to Nursing and Allied Health Literature; DOAJ, Directory of Open Access Journals; HINARI, Health Inter-Network Access to Research Initiative; WHO GIM, World Health Organization Global Index Medicus.

Out of the two thousand and twenty (2020) studies identified, a total of ninety-one (91) were included in the data analysis after quality appraisal, duplicate removal, screening, and data extraction (Fig. 1, Table S3) . The quality of the papers included in the review work was either good (∼89%) or fair (∼11%) (Table S3).

**Fig. 2:**
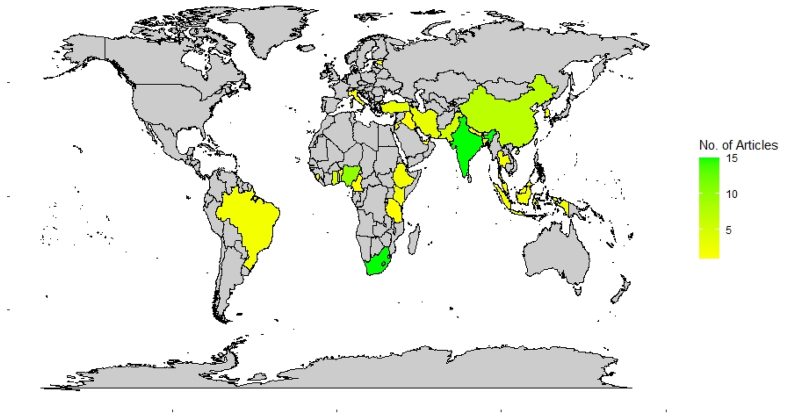
Geographical distribution of reviewed article origins by country.

The 91 peer-reviewed articles were published between the years 1982–2021 from 28 different countries and four continents (Fig. 2, Table 1). The four continents consisted of Asia (49.5%, 45/91), Africa (42.9%, 39/91), Europe (5.5%, 5/91), and South America (2.2%, 2/91) (Fig. S1). Africa and Asia accounted for ∼95.6% (84/91) of the peer-reviewed article under consideration. This is unsurprising since herbal medicines are more predominant in developing countries than other LMICs in Europe and South America [70,71]. Close to half (44.0%, n = 40) of the articles were together from studies conducted in South Africa (n = 15), India (n = 15), and Nigeria (n = 10), making Asia and Africa the dominant origins of these studies (Table 2). This correlates positively with the high prevalence of herbal medicines used in the above-listed countries [58,71].

**Table 1:** Reviewed articles per continent and country on contaminants in HMPs.

Country	Continent	Assay Method	Type of Contaminant	Reference
South Africa	Africa	Microbial Culture	Microbial	[72]
South Africa	Africa	AAS	Metal	[73]
Kingdom of Saudi Arabia	Asia	GC-MS	Metal	[74]
		ICP-MS	Metal	
		Microbial Culture	Microbial	
China	Asia	GC	Residual Solvent	[75]
Thailand	Asia	HPLC	Mycotoxins	[18]
South Africa	Africa	Voltammetry	Metal	[76]
South Africa	Africa	Microbial Culture	Microbial	[77]
Nigeria	Africa	AAS	Metal	[78]
India	Asia	AAS	Metal	[79]
South Africa	Africa	ICPS	Metal	[80]
China	Asia	GC	Pesticide	[81]
Nigeria	Africa	Microbial Culture	Microbial	[82]
India	Asia	GC	Pesticide	[83]
South Africa	Africa	HPLC	Mycotoxins	[84]
South Africa	Africa	ICPS	Metal	[85]
South Korea	Africa	HPLC	Mycotoxins	[86]
India	Asia	ICPS	Metal	[87]
India	Asia	AAS	Metal	[88]
Estonia	Europe	AAS	Metal	[89]
Pakistan	Asia	HPLC	Mycotoxins	[90]
India	Asia	AAS	Metal	[91]
India	Asia	GC	Metal	[92]
China	Asia	ICPS; AFS	Metal	[93]
India	Asia	AAS	Metal	[94]
		Microbial Culture	Microbial	
		GC	Pesticide	
Ghana	Africa	AAS	Metal	[95]
Nigeria	Africa	HPLC-MS	Mycotoxins	[96]
Kenya	Africa	Microbial Culture	Microbial	[97]
China	Asia	HPLC-MS	Mycotoxins	[98]
South Africa	Africa	ICPS	Metal	[99]
Jordan	Asia	AAS	Metal	[100]
India	Asia	GC	Residual Solvent	[101]
Bangladesh	Asia	Microbial Culture	Microbial	[102]
Turkey	Europe	ICPS	Metal	[103]
Malaysia	Asia	ICP-MS	Metal	[104]
Pakistan	Asia	AAS	Metal	[105]
Pakistan	Asia	AAS	Metal	[106]
Nigeria	Africa	Microbial Culture	Microbial	[107]
		TLC	Mycotoxins	
Iran	Asia	Microbial Culture	Microbial	[108]
Pakistan	Asia	AAS	Metal	[109]
South Africa	Africa	Microbial Culture	Microbial	[20]
Kingdom Of Saudi Arabia	Asia	AAS	Metal	[110]
		Microbial Culture	Microbial	
China	Asia	UPLC-MS	Mycotoxins	[111]
United Arab Emirates	Asia	AAS	Metal	[112]
Sierra Leone	Africa	Microbial Culture	Microbial	[113]
Iran	Asia	ICP-MS	Metal	[114]
		ICPS		
Brazil	South America	ICP-MS	Metal	[115]
India	Asia	AAS	Metal	[116]
Bangladesh	Asia	AAS	Metal	[117]
		Microbial Culture	Microbial	
India	Asia	Microbial Culture	Microbial	[118]
		TLC & HPLC	Mycotoxins	
South Africa	Africa	Microbial Culture	Microbial	[119]
South Africa	Africa	ICPS	Metal	[120]
Pakistan	Asia	HPLC	Mycotoxins	[121]
Ghana	Africa	AAS	Metal	[122]
Kingdom Of Saudi Arabia	Asia	AAS	Metal	[123]
Turkey	Europe	HPLC	Mycotoxins	[19]
Tanzania	Africa	Microbial Culture	Microbial	[21]
Ethiopia	Africa	Microbial Culture	Microbial	[124]
Kenya	Africa	INAA	Metal	[125]
India	Asia	ICP-MS	Metal	[126]
India	Asia	HPLC	Mycotoxins	[127]
South Africa	Africa	ICP-MS	Metal	[128]
Thailand	Asia	AAS	Metal	[129]
Nigeria	Africa	Microbial Culture	Microbial	[130]
Benin	Africa	HPLC	Mycotoxins	[131]
South Africa	Africa	ICPS	Metal	[132]
India	Asia	AAS	Metal	[133]
		GC	Pesticide	
China	Asia	ICP-MS	Metal	[134]
Turkey	Europe	ICPS	Metal	[135]
China	Asia	FAPS	Mycotoxins	[136]
Palestine	Asia	AAS	Metal	[137]
South Africa	Africa	ICPS	Metal	[27]
Nigeria	Africa	Microbial Culture	Microbial	[138]
Iran	Asia	ICPS	Metal	[139]
India	Asia	GC	Residual Solvent	[140]
South Africa	Africa	ICP-MS	Metal	[141]
Cameroon	Africa	Microbial Culture	Microbial	[142]
Nigeria	Africa	ICPS	Metal	[143]
Nigeria	Africa	AAS	Metal	[144]
Ethiopia	Africa	ICP-MS	Metal	[145]
Iraq	Asia	HPLC	Microbial	[146]
		HPLC	Mycotoxins	
Italy	Europe	Microbial Culture	Microbial	[147]
Brazil	South America	Microbial Culture	Microbial	[35]
Nepal	Asia	AAS	Metal	[148]
Nigeria	Africa	Immunoassay	Mycotoxins	[149]
Indonesia	Asia	AAS	Metal	[150]
		Microbial Culture	Microbial	
India	Asia	AAS	Metal	[151]
Ghana	Africa	AAS	Metal	[152]
		Microbial Culture	Microbial	
Indonesia	Asia	Microbial Culture	Microbial	[153]
		HPLC	Mycotoxins	
Nigeria	Africa	ICP-MS	Metal	[154]
Ghana	Africa	GC-MS	Pesticide	[24]
Ghana	Africa	GC	Residual Solvent	[22]

AAS, Atomic absorption spectroscopy; FAPS, Fluorescent Aptasensor PicoGreen-Based Strategy; GC, Gas chromatography; GC-MS, Gas chromatography mass spectrometry; ICP-MS, Inductively coupled plasma mass spectrometry; ICPS, Inductively coupled plasma spectroscopy; HPLC, High performance liquid chromatography; AFS, Atomic fluorescence spectrometry; HPLC-MS, High performance liquid chromatography mass spectrometry; INAA, Instrumental neutron activation analysis; TLC, Thin-layer chromatography; UPLC-MS, Ultra performance liquid chromatography mass spectrometry.

**Table 2:** Types of contaminants analyzed in each of the reviewed articles per continent and country.

		Contaminant				
	Number of Articles	Metal (n = 51)	Microbial (n = 26)	Mycotoxin (n = 17)	Pesticide (n = 5)	Residual Solvent (n = 4)
Continent						
Africa	39	19 (48.7)	14 (35.9)	6 (15.4)	1 (2.6)	1 (2.6)
Asia	45	28 (62.2)	10 (22.2)	10 (22.2)	4 (8.9)	3 (6.7)
Europe	5	3 (60.0)	1 (20.0)	1 (20.0)	0 (0.0)	0 (0.0)
South America	2	1 (50.0)	1 (50.0)	0 (0.0)	0 (0.0)	0 (0.0)
**Country**						
Bangladesh	2	1 (50.0)	2 (100.0)	0 (0.0)	0 (0.0)	0 (0.0)
Benin	1	0 (0.0)	0 (0.0)	1 (100.0)	0 (0.0)	0 (0.0)
Brazil	2	1 (50.0)	1 (50.0)	0 (0.0)	0 (0.0)	0 (0.0)
Cameroon	1	0 (0.0)	1 (100.0)	0 (0.0)	0 (0.0)	0 (0.0)
China	7	2 (28.6)	0 (0.0)	3 (42.9)	1 (14.3)	1 (14.3)
Estonia	1	1 (100.0)	0 (0.0)	0 (0.0)	0 (0.0)	0 (0.0)
Ethiopia	2	1 (50.0)	1 (50.0)	0 (0.0)	0 (0.0)	0 (0.0)
Ghana	5	3 (60.0)	1 (20.0)	0 (0.0)	1 (20.0)	1 (20.0)
India	15	10 (66.7)	2 (13.3)	2 (13.3)	3 (20.0)	2 (13.3)
Indonesia	2	1 (50.0)	2 (100.0)	1 (50.0)	0 (0.0)	0 (0.0)
Iran	3	2 (66.7)	1 (33.3)	0 (0.0)	0 (0.0)	0 (0.0)
Iraq	1	0 (0.0)	1 (100.0)	1 (100.0)	0 (0.0)	0 (0.0)
Italy	1	0 (0.0)	1 (100.0)	0 (0.0)	0 (0.0)	0 (0.0)
Jordan	1	1 (100.0)	0 (0.0)	0 (0.0)	0 (0.0)	0 (0.0)
Kenya	2	1 (50.0)	1 (50.0)	0 (0.0)	0 (0.0)	0 (0.0)
Kingdom Of Saudi Arabia	3	3 (100.0)	2 (66.7)	0 (0.0)	0 (0.0)	0 (0.0)
Malaysia	1	1 (100.0)	0 (0.0)	0 (0.0)	0 (0.0)	0 (0.0)
Nepal	1	1 (100.0)	0 (0.0)	0 (0.0)	0 (0.0)	0 (0.0)
Nigeria	10	4 (40.0)	4 (40.0)	3 (30.0)	0 (0.0)	0 (0.0)
Pakistan	5	3 (60.0)	0 (0.0)	2 (40.0)	0 (0.0)	0 (0.0)
Palestine	1	1 (100.0)	0 (0.0)	0 (0.0)	0 (0.0)	0 (0.0)
Sierra Leone	1	0 (0.0)	1 (100.0)	0 (0.0)	0 (0.0)	0 (0.0)
South Africa	15	10 (66.7)	4 (26.7)	1 (6.7)	0 (0.0)	0 (0.0)
South Korea	1	0 (0.0)	0 (0.0)	1 (100.0)	0 (0.0)	0 (0.0)
Tanzania	1	0 (0.0)	1 (100.0)	0 (0.0)	0 (0.0)	0 (0.0)
Thailand	2	1 (50.0)	0 (0.0)	1 (50.0)	0 (0.0)	0 (0.0)
Turkey	3	2 (66.7)	0 (0.0)	1 (33.3)	0 (0.0)	0 (0.0)
United Arab Emirates	1	1 (100.0)	0 (0.0)	0 (0.0)	0 (0.0)	0 (0.0)

A total of eight thousand and thirty-seven (8037) data observations were extracted from the 91 peer-reviewed articles included in this systematic review. Out of the 8037 data observations, 7518 had reported mean contaminant concentration levels and the reported contaminants' regulatory limits. Hence, the 7518 data observations were used in the subsequent data analysis. The data analysis did not include all data observations without contaminant concentration and regulatory limits.

**Fig. 3:**
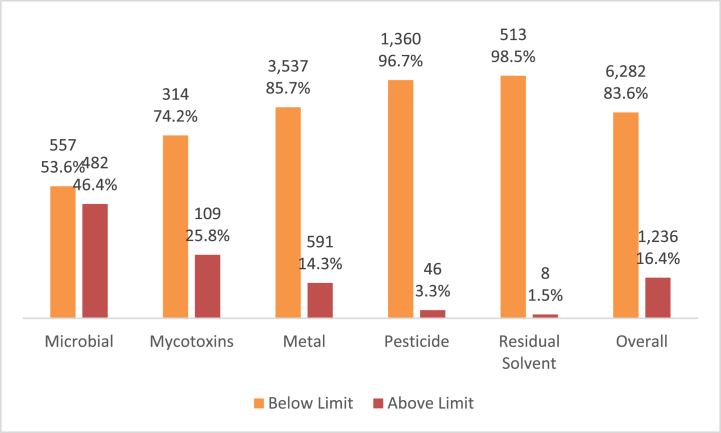
Contaminant levels reported in the reviewed articles.

Overall, 16.4% (1236/7518) of the samples had contaminant levels above the regulatory limit, whilst 83.6% (6282/7518) were within the regulatory limits (Fig. 3). Samples tested for microbial contaminants had the highest proportion (46.4%, 482/1039) of contaminants exceeding the regulatory limit, followed by mycotoxins (25.8%, 109/423) and metals (14.3%, 591/4128). However, samples tested for residual solvent contaminants (1.5%, 8/521) recorded the least proportion of samples with levels exceeding the regulatory limit (Fig. 3).

## 4 Contaminants

Contaminants are usually added unintentionally to herbal products, food, or water [155]. The most expected contaminants found in medicinal herbs and herbal products are biologicals (bacteria and fungi) and chemicals (metals, mycotoxins, pesticides, and residual solvents) [15].

The different contaminants analyzed in the articles were metals, microbial, mycotoxins, pesticides, and residual solvents (Table 1), which agrees with the existing literature [15]. Each study assessed a minimum of 1 and a maximum of 3 contaminants (Fig. S2). Fig. S1 shows the distribution of contaminants evaluated in the 91 studies. Metals (56.0%, 51/91) were the commonest type of contaminant measured in the 91 peer-reviewed articles, while pesticides (5.5%, 5/91) [24,[81], [83], [94], [133]] and residual solvents (4.4%, 4/91) [22,[75], [101], [140]] were the least type of contaminants assessed (Fig. S3).

Of the 39 articles from Africa, almost half of them tested for metals (48.7%, 19/39) and 35.9% (14/39) of them tested for microbial contaminants, while 15.4% (6/39) of them also tested for mycotoxins. Pesticides [24] and residual solvents [22] were assessed in only one peer-reviewed article each. In studies from Asia, 62.2% (28/45) of them tested for metals, while about a little more than one-fifth of them also tested for the presence of microbial (22.2%, 10/45) and mycotoxins (22.2%, 10/45). In Europe, three out of the five studies reviewed measured metals [[89], [103], [135]], while the remaining two papers tested for either microbial [147] or mycotoxins [19]. South America had only two studies, each measuring metals [115] and microbial contaminants [35]. None of the studies from South America measured mycotoxins, pesticides, and residual solvents. Details of the types of contaminants assessed in each of the reviewed articles per continent and country are shown in Table 2.

### 4.1 Metals

Elemental impurities (metals) are traces of metals that may be present in orthodox or herbal products and may occur naturally, be added intentionally, or be introduced inadvertently [60]. Elemental impurities are usually classified as essential macro, essential micro, and non-essential metals [156].

Among the 51 peer-reviewed articles that measured metals, 11.8% (6/51), 72.5% (37/51), and 96.1% (49/51) tested for essential macro, essential micro, and non-essential metals, respectively (Table 3). Essential macro metals were measured in studies from only Asia and South America, while essential micro metals were found in studies from all the continents except South America. Although studies from all four continents tested for non-essential metals, the majority, 45/49 (91.8%), were from Africa and Asia.

**Table 3:** The subtype of contaminants analyzed in each of the reviewed articles per continenta).

	Continent				
	Africa	Asia	Europe	South America	Total
Metals					
Essential Macro	5 (26.3)	0 (0.0)	1 (33.3)	0 (0.0)	6
Essential Micro	15 (78.9)	20 (71.4)	2 (66.7)	0 (0.0)	37
Non-Essential	17 (89.5)	28 (100.0)	3 (100.0)	1 (100.0)	49
**Microbial**					
Bacteria	13 (92.9)	4 (44.4)	1 (100.0)	1 (100.0)	19
Fungi	6 (42.9)	7 (77.8)	0 (0.0)	0 (0.0)	13
Not Indicated	3 (21.4)	2 (22.2)	0 (0.0)	0 (0.0)	5
**Mycotoxins**					
Aflatoxin	5 (83.3)	10 (100.0)	1 (100.0)	0 (0.0)	16
Fumonisins	3 (50.0)	1 (10.0)	0 (0.0)	0 (0.0)	4
Ochratoxin	2 (33.3)	3 (30.0)	0 (0.0)	0 (0.0)	5
Patulin	0 (0.0)	2 (20.0)	0 (0.0)	0 (0.0)	2
Trichothecenes	2 (33.3)	0 (0.0)	0 (0.0)	0 (0.0)	2
Zearalenone	2 (33.3)	0 (0.0)	0 (0.0)	0 (0.0)	2
**Pesticide**					
Organochlorine	1 (100.0)	4 (100.0)	0 (0.0)	0 (0.0)	5
Organophosphate	1 (100.0)	1 (25.0)	0 (0.0)	0 (0.0)	2
Pyrethroid	1 (100.0)	0 (0.0)	0 (0.0)	0 (0.0)	1
**Residual Solvent**					
Class 1	1 (100.0)	1 (33.3)	0 (0.0)	0 (0.0)	2
Class 2	1 (100.0)	3 (100.0)	0 (0.0)	0 (0.0)	4
Class 3	1 (100.0)	3 (100.0)	0 (0.0)	0 (0.0)	4
Not Classified	0 (0.0)	2 (66.7)	0 (0.0)	0 (0.0)	2

a) The denominator for each cell is the number of articles that measured that type of contaminant, as stated in Table 2.

#### 4.1.1 Essential macro metals

Natural elements required by the body in large quantities are known as essential macro elements, which include K, Ca, Mg, Cl, and P [157]. Essential macro metal elements play critical roles such as homeostasis, absorption, transport, storage, and excretion in the human body [158]. However, high levels of these elements may cause severe malfunctioning of the body and death in some instances since these macro elements directly affect the metabolism and physiology of the human body [32].

Three essential macro metals, namely, Ca, K, and Mg were reported in 6 studies. Two-thirds of the papers (66.7%) reported on Ca and Mg, while 50% (3/6) reported on K (Table S4). Fortunately, all the reported essential macro metals (K and Mg) levels were below their regulatory limits (Table S4). Notwithstanding, essential macro elements should be monitored constantly in HMPs due to their potential toxic effects on the human body [32].

#### 4.1.2 Essential micro metals

The essential micro metals, which include B, Co, Cr, Cu, F, Fe, I, Mn, Mo, Na, Ni, Se, Si, V, and Zn, are micronutrients needed by the human body in minimal quantities for growth, development, and physiology of the human body [156,157]. However, high levels of these metals in humans may cause adverse effects such as liver and kidney problems, abdominal pain, cramps, nausea, diarrhea, vomiting, hyperthyroidism, psychologic and neurologic disorder, hair and nail loss, and brittleness, skin rash, garlic breath odor, and nervous system abnormalities [33].

Twelve essential micro metals were identified in 37 studies: B, Co, Cr, Cu, Fe, Mn, Mo, Na, Ni, Se, Si, and Zn. The predominant essential micro metals identified were Zn (81.1%, 30/37), Cu (78.4%, 29/37), Mn (70.3%, 26/37), Cr (67.6%, 25/37), and Fe (64.9%, 24/37) (Table S4). The proportion of essential micro metals, namely, Fe, Mn, Zn, and Cu, that exceeded the required regulatory limits were 8.5% (31/364), 4.1% (15/370), 3.9% (16/413) and 0.7% (3/419), respectively (Table S4).

Since some HMPs are taken over a long duration [159], toxicity from essential micro metals such as Fe may occur, leading to serious health problems and even death [156]. Specifically, Fe toxicity adversely affects the gastrointestinal, cardiovascular, and central nervous systems and the kidney, liver, and blood [33]. Prolonged use of HMPs contaminated with Fe may have profound health implications for patients with hemochromatosis [33] and individuals with anemia due to hemoglobin synthesis abnormalities [33]. Contamination of HMPs with Mn is a serious health concern to consumers since it can lead to Mn toxicity. Mn toxicity commonly results in neurotoxicity (i.e., manganism), characterized by irritability, tremors, hallucinations, difficulty walking, aggressiveness, and facial muscle spasms [33,156]. Although Zn is typically considered relatively nontoxic, gastrointestinal disorders have been reported for Zn toxicity [33,156]. However, prolonged intake of high levels of Zn can negatively affect Cu uptake in the body, causing Zn-induced myeloneuropathy [33,156]. This poses a severe public health concern for consuming HMPs contaminated with Zn. Long-term consumption of HMPs with high levels of Cu may lead to Cu toxicity, which may cause diarrhea, headaches, and severe health effects such as liver, kidney, heart, and brain damage, and may also lead to death [33]. Medical conditions such as Wilson's disease, Menkes disease, liver disease, hepatitis, Hodgkin lymphoma, leukemia, brain cancer, liver cancer, breast cancer, and diabetes, in which the excretion of Cu is compromised, and can lead to Cu toxicity after prolonged use of Cu contaminated HMPs [156,160,161]. Consequently, Fe, Mn, Zn, and Cu levels should be monitored by National Regulatory Authorities (NRAs) before HMPs approval and further active post-market surveillance after product approval.

However, Co, Cr, Mo, and Se levels were all within their respective regulatory limits (Table S4).

#### 4.1.3 Non-essential metals

Metals that have no functional biological activity in human cells and are also toxic in even minute quantities are known as non-essential [162], which include As, Ba, Cd, Pb, Hg, Ag, Se, Sn, and Al [28,34,156,162,163]. But Sn has also been described as a possible essential trace element since it participates in necessary daily physiological activities of the human body [32,164]. The most predominant non-essential metals of interest are As, Cd, Cr, Pb, and Hg [165]. Non-essential metals threaten human health, and adverse health effects have been reported [34].

Thirteen non-essential metals, namely, Ag, Al, As, Ba, Bi, Cd, Hg, Pb, Rb, Sn, Sr, U, and V were reported in 49 studies. Most of the studies identified Pb (87.8%, 43/49), Cd (77.6%, 38/49), As (49%, 24/49), and Hg (38.8%, 19/49) as the non-essential metals (Table S4). This is consistent with a previous literature review study [163] since most studies on elemental impurities focus on these non-essential metals [28,34,156,162,163].

More than half of the samples with their average contaminant levels and their regulatory limits reported for Pb (57.6%, 377/655) exceeded their regulatory limits (Table S4). Although Pb occurs naturally in the environment, high levels are found in nature due to anthropogenic activities [163]. This may account for the high concentration levels of Pb in most of the HMPs from the various studies included in this systematic review. This is a serious health concern since Pb has been identified as a potential human carcinogen [28], and in some studies, it has been linked to an increased incidence of stomach, lung, and bladder cancers [34]. Also, Pb can affect most human organs and systems, such as cardiovascular and blood. Pb can cause adverse health effects such as neurotoxicity and nephrotoxicity [28,34,156,162,163]. Exposure to high levels of Pb may result in serious adverse health issues such as miscarriage in women, poor sperm production in men, and, ultimately death [28].

Also, the proportion of samples that had their average contaminant levels above the regulatory limit was 18.3% (88/480), 10.7% (24/225), and 11.3% (29/257) for Cd, Hg, and As, respectively (Table S4).

Cd is a very toxic metal and occurs naturally in the environment but is found predominantly in soils, rocks, and industrial and agricultural sources [28,156]. Cd is a known human carcinogen, and there have been increased cancer risks, such as lung cancer and mortality in environmentally exposed populations [28,156,166]. Severe lung damage and gastrointestinal disorders due to ingestion of high quantities of Cd have been reported [28,166]. Also, kidney disease, lung damage, and fragile bones have been associated with long-term exposure to lower quantities of Cd in human cells and sometimes result in death [28,156,166]. Regular consumption of HMPs contaminated with Cd has profound public implications, especially for tobacco smokers since they experience almost twice Cd exposure as nonsmokers [166].

Although Hg is naturally found in the environment, it is mainly due to anthropogenic activities [26]. Also, mining ore deposits, burning coal and waste, and some manufacturing plants are significant sources of inorganic Hg to the environment. Hg-based fungicides, natural deposits, and refuse dumps are also substantial sources of Hg in water bodies and soil [166]. Mercuric chloride and methylmercury are potential human carcinogens with high exposure levels leading to damage to the brain, kidneys, and developing fetuses permanently. Hg affects the nervous system, especially the brain, which may result in irritability, shyness, tremors, changes in vision or hearing, and memory problems. Short-term exposure to high levels of metallic mercury vapors may cause lung damage, gastrointestinal disturbances, increases in blood pressure or heart rate, skin rashes, and eye irritation [26,28,156].

Arsenic (As) may be released in larger quantities through volcanic activity, erosion of rocks, forest fires, and anthropogenic environmental activities apart from natural occurrences [28]. Arsenic is a known human carcinogen that can cause skin, lung, liver, and bladder cancer. Exposure to low levels of arsenic can result in gastrointestinal disorders, reduced levels of blood cells, abnormal heart rhythm, damage to blood vessels, and numbness of hands and feet. However, ingestion of very high levels can result in death. Long-term exposure to low levels of As can cause skin disorders [28].

On the other hand, Ag (100%, 26/26) and Sn (100%, 26/26) had their limits within the regulatory requirements (Table S4).

### 4.2 Microbial

The microbial contaminants indicated in 26 peer-reviewed articles were bacteria (73.1%, 19/26) and fungi (50.0%, 13/26). However, 19.2% (5/26) did not specify the type of microbial contaminant measured (Table 3). African studies mostly tested for bacteria (92.9%, 13/14) and fungi (42.9%, 6/14). For studies from Asia that tested for microbial contaminants, most of them tested for the presence of fungi (77.8%, 7/9) and bacteria (44.4%, 4/9). Single studies from Europe [147] and South America [35] tested for only bacteria as the sub-type of microbial contaminants (Table 3).

#### 4.2.1 Bacteria

Fifty-seven different bacteria species were reported in 19 studies, while the type of bacteria species was not reported in three studies (Table S5). The bacteria species included *Acinetobacter baumannii*, *Acinetobacter calcoaceticus*, *Acinetobacter lwoffii*, *Acinetobacter species*, *Aerobic bacteria*, *Bacillus amyloliquefaciens*, *Bacillus cereus*, *Bacillus lentus*, *Bacillus megaterium*, *Bacillus polymyxa*, *Bacillus pumilus*, *Bacillus species*, *Bacillus subtilis*, *Bacillus vallismortis*, *Citrobacter diversus*, *Citrobacter intermidius*, *Citrobacter species*, *Clostridium species*, Coliforms, *Corynebacterium pseudodiphtheriticum*, *Corynebacterium xerosis*, *Enterobacter aerogenes*, *Enterobacter cloacae*, *Enterobacter species*, *Enterobacteria species*, *Enterococcus species*, *Escherichia coli*, *Klebsiella oxytoca*, *Klebsiella ozaenae*, *Klebsiella pneumoniae*, *Lactobacillus casei*, *Leclercia adecarboxylata*, *Listeria grayi*, *Listeria monocytogenes*, *Listeria murrayi*, *Micrococcus luteus*, *Pantoea species*, *Proteus vulgaris*, *Providencia species*, *Providencia stuartii*, *Pseudomonas aeruginosa*, *Pseudomonas cetrimide*, *Pseudomonas oryzihaitans*, *Pseudomonas species*, *Salmonella species*, *Serratia marcescens*, *Serratia species*, *Shigella dysenteriae*, *Shigella species*, *Sphingomonas paucimobilis*, *Staphylococcus aureus*, *Staphylococcus epidermidis*, *Staphylococcus saprophyticus*, *Staphylococcus species*, *Streptococcus faecalis*, *Streptococcus mitis*, *Streptococcus pyogenes*, and *Streptococcus species*. The commonest bacteria species found were *Escherichia coli* (52.3%, 10/19) and *Salmonella species* (42.1%, 8/19). Aerobic bacteria (21.1%, 4/19), *Bacillus species* (26.3%, 5/19), *Pseudomonas aeruginosa* (21.1%, 4/19), and *Staphylococcus aureus* (26.3%, 5/19) were also reported in about one-fifth to a quarter of the studies.

Among the bacteria species, the following had all their reported levels above the regulatory limits (Table S5); *Acinetobacter calcoaceticus*, *Acinetobacter species*, *Bacillus cereus*, *Bacillus polymyxa*, *Bacillus pumilus*, *Citrobacter diversus*, *Citrobacter intermidius*, *Citrobacter species*, *Clostridium species*, *Corynebacterium pseudodiphtheriticum*, *Corynebacterium xerosis*, *Enterobacter aerogenes*, *Enterobacter species*, *Enterococcus species*, *Klebsiella ozaenae*, *Lactobacillus casei*, *Listeria grayi*, *Listeria monocytogenes*, *Listeria murrayi*, *Micrococcus luteus*, *Proteus vulgaris*, *Providencia species*, *Providencia stuartii*, *Serratia marcescens*, *Shigella dysenteriae, Staphylococcus epidermidis*, and *Streptococcus pyogenes*.

The proportion of bacteria species, namely, *Bacillus megaterium*, *Bacillus subtilis*, *Enterobacter cloacae, Enterobacteria species*, *Escherichia coli*, *Klebsiella pneumoniae*, *Pseudomonas aeruginosa*, *Salmonella species*, *Serratia species*, *Staphylococcus aureus*, *Staphylococcus saprophyticus*, and *Staphylococcus species* that exceeded the required regulatory limits were 71.4% (5/7), 92.9% (13/14), 91.7% (11/12), 83.3% (10/12), 57.7% (45/78), 93.8% (15/16), 72.2% (13/18), 51.7% (31/60), 80.0%. (4/5), 68.4% (26/38), 66.7% (2/3), and 83.3% (10/12), respectively (Table S5). However, *Aerobic bacteria*, *Bacillus species*, Coliforms, *Shigella species*, *Streptococcus faecalis*, and *Streptococcus species* had >50% of their reported levels below the required regulatory limits (Table S5).

On the other hand, *Acinetobacter baumannii*, *Acinetobacter lwoffii*, *Bacillus amyloliquefaciens*, *Bacillus lentus*, *Bacillus vallismortis*, *Klebsiella oxytoca*, *Leclercia adecarboxylata, Pantoea species*, *Pseudomonas cetrimide*, *Pseudomonas oryzihaitans*, *Pseudomonas species*, *Sphingomonas paucimobilis*, and *Streptococcus mitis* had all their reported levels below the regulatory limits (Table S5).

#### 4.2.2 Fungi

Thirty different fungi species were reported in 13 studies (Table S5). The reported fungi species were *Actinomadura madurae*, *Alternaria species*, *Aspergillus flavus*, *Aspergillus fumigatus*, *Aspergillus nidulans*, *Aspergillus niger*, *Aspergillus oryzae*, *Aspergillus species*, *Candida albicans*, *Candida pseudotropicalis*, *Candida species*, *Candida tropicalis*, *Cladosporium species*, *Cryptococcus neoformans*, *Curvularia species*, *Fungi species*, *Fusarium species*, *Geotricum species*, *Hansenula anomala*, *Madurella mycetomatis*, Moulds, *Mucor species*, *Penicillium species*, *Rhizopus species*, *Rhodotorula glutinis*, *Saccharomyces cerevisiae*, *Torulopsis candida*, *Torulopsis glabrata*, *Trichoderma harzianum*, and *Trichosporon cutaneum*. Moulds, *Penicillium species*, *Mucor species*, *Aspergillus niger,* and *Aspergillus flavus* were reported in about a quarter of the studies.

All the reported levels of the following fungi species: *Actinomadura madurae*, *Aspergillus nidulans*, *Aspergillus oryzae*, *Candida pseudotropicalis*, *Candida tropicalis*, *Cryptococcus neoformans*, *Hansenula anomala*, *Madurella mycetomatis*, *Rhodotorula glutinis*, *Saccharomyces cerevisiae*, *Torulopsis candida*, *Torulopsis glabrata*, *Trichoderma harzianum*, and *Trichosporon cutaneum* were above the established regulatory limits (Table S5).

On the other hand, all the reported levels of the fungi species *Alternaria species*, *Candida species*, *Cladosporium species*, *Curvularia species*, *Fusarium species*, *Geotricum species*, *and Rhizopus species* were below the published regulatory limits (Table S5).

The proportion of fungi species, namely, *Candida albicans* and Moulds, that exceeded the required regulatory limits were 88.9% (8/9), and 82.1% (23/28), respectively (Table S5). However, *Aspergillus flavus*, *Aspergillus fumigatus*, *Aspergillus niger*, *Aspergillus species*, *Fungi species*, *Mucor species*, and *Penicillium species* had >50% of their reported levels below the required regulatory limits (Table S5).

The overall microbial findings showed that even though only a few (16.4%) of contaminant presence in HMPs were above regulatory limits, microbial contaminants were the highest in the proportion of 46.4% exceeding the regulatory limits, followed by mycotoxins and metals in LMICs Fig. 2. Evidence from African and Asian LMICs such as Ghana [167], Tanzania [21], and India [168] attribute microbial contamination of HMPs to pollution in the chain of production, from harvested raw materials used in the preparation, handling, processing, storage, and transportation. Other factors such as the use of untreated water supply, poor quality of packaging materials, use of contaminated containers, working from polluted fecal environments, or poor personal hygiene behaviors during handling have been reported as potential sources of microbial contamination of HMPs from these settings [21,167,168]. Poor microbial quality of HMPs implies that they may serve as routes for transmitting pathogenic microbial agents [169]. The presence of microbial contaminants beyond regulatory limits could constitute serious health risks to consumers and patients by serving as an additional source of infection to the pre-existing medical condition for which the HMPs were initially indicated [35].

Most microbial contaminants identified in HMPs were of bacterial origin from African LMICs studies compared with fungi from Asia, with the least bacteria contamination reported from Europe and South America (Table 3). The commonest bacteria species reported were *Escherichia coli* and *Salmonella species*. The prevailing tropical and sub-tropical climatic patterns in many parts of Africa and Asia might provide favorable warm and humid conditions for the survival and multiplication of microbial agents, unlike the temperate conditions predominant in Europe and the Americas [170,171]. In addition, the relatively weak sanitation and waste management systems and high defecation rates in open spaces enable the persistence of microbial growth in such environments from which raw materials for HMPs are harvested in Africa [172]. Insufficient hygiene practices along the HMP production chain might also expose the products to the hazard of bacterial contamination [173].

This review reported that almost 90% of *Candida albicans* and more than 80% of moulds exceeded the required regulatory limits (Table S5). *Candida albicans* are responsible for most human infections caused by pathogenic fungi [174]. Even though the species is a normal flora that colonizes the oral, gastrointestinal, and genital tracts, they become important pathogens when there is abrasion of barrier integrity, reduced host immune responses, acquisition of different virulence traits, or access into deeper tissues of both immune-competent and immune-compromised person [175]. Pathogenic *Candida albicans* in HMPs can cause diseases on the skin, in the bloodstream, and in internal organs such as the brain and spinal cord, liver, spleen, heart, and kidneys, with associated mortality among consumers [174]. The presence of moulds such as *Aspergillus niger*, *Aspergillus flavus*, and other *Aspergillus species*, known as mycotoxin producers, may pose severe health risks when present in orally used HMPs [176,177]. The severity of mycotoxin-related adverse health effects on consumers depends on the toxicity, degree of exposure, age, and nutritional status of the individual, and the possible synergistic effects of other chemical agents to which they are exposed [178]. They are known to have teratogenic and carcinogenic effects, cause liver damage, reduce immunity, and impair fertility [[176], [177], [178], [179]]. They also cause brain necrosis and medical complications of kidney and gastrointestinal cancer. For example, concomitant exposure to aflatoxins produced by Aspergillus flavus and Hepatitis B infection has been reported to aggravate the clinical prognosis of hepatocellular carcinoma in African populations [179].

The bacteria species, namely, *Bacillus megaterium*, *Bacillus subtilis*, *Enterobacter cloacae*, *Enterobacteria species*, *Escherichia coli*, *Klebsiella pneumoniae*, *Pseudomonas aeruginosa*, *Salmonella species*, *Serratia species*, *Staphylococcus aureus*, *Staphylococcus saprophyticus*, and *Staphylococcus species* exceeded the required regulatory limits, out of the 26 peer-reviewed articles that reported microbial contamination (Table S5). According to the guidelines of the WHO [61] and the European pharmacopoeia [180], *Salmonella species* and other coliforms, such as *E. coli,* must not be present in HMPs intended for internal use in humans at any stage. These microbial agents indicate fecal contamination from poor personal hygiene and handling practices during the production chain [61,181]. The presence of *E. coli* does imply not only fecal contamination but also the potential presence of other, more virulent, strains such as the Shiga toxin-producing *E. coli*, capable of causing life-threatening diseases including hemolytic uraemic syndrome, particularly in young children and elderly consumers of contaminated HMPs, living in LMICs [182]. The presence of *Bacillus species* may be due to the inability to remove bacterial spores during cleaning and handling processes in the production chain. The persistence of these spores in the soil may contaminate the raw materials used in HMP production. Specifically, toxins produced by *Bacillus cereus* have also been reported to cause toxigenic diarrhea when ingested in contaminated HMPs in African LMICs [77]. Different *Staphylococcal species* have been previously reported to contaminate HMPs circulating in many LIMCs, including Ghana [167], Tanzania [21], Nigeria [173], and India [168]. Their presence is highly related to using unhygienic equipment and poor personal hygiene practices in handling HMPs [173]. *Staphylococcus aureus*, for example, is an important cause of food-borne intoxication following ingestion of its preformed heat-resistant enterotoxins that results in severe gastroenteritis, in addition to the toxic shock and staphylococcal scalded skin syndrome outcomes [183,184]. *Pseudomonas aeruginosa* and *Klebsiella pneumoniae* contaminant presence, on the other hand, suggest improper washing and handling of raw materials used in preparing HMPs, and both have been implicated in causing urinary tract and severe respiratory diseases when ingested [167,185].

### 4.3 Mycotoxins

Mycotoxins are toxic secondary metabolites naturally produced by moulds, which cause disease and sometimes death in humans [186]. They are usually found in hot and humid climatic regions. They can directly contaminate herbal plants and their parts during pre- and post-harvest processing, transportation, and storage of herbal medicines [187]. Mycotoxins of public health concern to humans include aflatoxins, ochratoxin A, patulin, fumonisins, zearalenone, and nivalenol/deoxynivalenol, although over a hundred different types have been identified [186]. Mycotoxins pose acute and chronic health challenges such as liver cancer, reduced immunity, alterations in protein metabolism, gangrene, convulsions, and respiratory problems, among others, to humans [36].

Seventeen peer-reviewed articles measured either one or more of the following mycotoxins: aflatoxins, fumonisins, ochratoxins, patulin, trichothecenes, and zearalenone, which are the most predominant mycotoxins of public health concerns [186]. Mycotoxins were measured in all the studies from the four continents except South America. The most predominantly measured mycotoxins were aflatoxins (94.1%, 16/17). This is not surprising since aflatoxins are amongst the most poisonous mycotoxins and therefore are commonly monitored [188]. Africa, Asia, and Europe measured 6/7, 4/7, and 1/7 of the different types of mycotoxins, respectively (Table 3).

#### 4.3.1 Aflatoxins

Aflatoxins are produced by species of fungi, including *Aspergillus*, *Fusarium,* and *Penicillium*, but *A. flavus* and *A. parasiticus* are the commonest [186]. Aﬂatoxins have been studied extensively since it is the most harmful of all mycotoxins [118]. The classification of aflatoxins as either B1, B2, G1, or G2 is based on UV light color for their fluorescence (blue for B1 and B2; green for G1 and G2) and relative chromatographic mobility during thin-layer chromatography [186]. Aflatoxins B1, B2, G1, and G2 are the most common and harmful to humans, with the order of toxicity being B1 > G1 > B2 > G2 [189]. However, over twenty different types of aflatoxins have been reported [190].

The presence of aflatoxins in herbal medicines can pose acute and chronic health risks. Aﬂatoxins are toxic carcinogens, teratogens, and mutagens. They are classiﬁed as Group 1 human carcinogens by the International Agency for Research on Cancer as it is a cause of human primary hepatocellular carcinoma [191]. It is estimated that about 500 million people from developing countries are exposed to high levels of aflatoxins [192], increasing morbidity and mortality. Aflatoxins B1, B2, B3, G1, G1 + G2, G2, and G3 and total aflatoxins were reported in 16 peer-reviewed articles. Aflatoxins B1 (87.5%, 14/16), B2 (68.6%, 11/16), G1 (62.5%, 10/16), and G2 (43.8%, 7/16) were the most reported (Table S6), which is consistent with the literature [190].

The reported levels of aflatoxin B1 (27.7%, 52/188) and total aflatoxins (18.2%, 8/44) were above the required regulatory limits, while aflatoxin G1 (100%, 38/38), aflatoxins G1 + G2 (100%, 6/6), and aflatoxin G2 (100%, 30/30) were all within the required regulatory limits (Table S6).

#### 4.3.2 Fumonisins

Fusarium species produce fumonisins and consist mainly of fumonisin B1 and fumonisin B2. Fumonisins are probable human carcinogens implicated in oesophageal cancers [186]. Also, they have been shown as hepatotoxic, nephrotoxic, atherogenic, immunosuppressive, and embryotoxic in animal studies [193]. Acute exposure to fumonisin B1 resulted in gastrointestinal disorders [194].

Among the four studies that reported on fumonisins, 75% (3/4) measured fumonisins B1 while 25% (1/4) measured fumonisins B2 (Table S6). However, 25% (1/4) did not indicate the subtype of measured fumonisins. The proportion of fumonisins B1 and B2 that exceeded the required regulatory limits were 93.9% (31/33), and 100% (2/2), respectively. This is a severe public health concern due to the above-indicated risk factors for fumonisins. Subsequently, there is a need for continuous monitoring of fumonisins in HMPs. However, all the reported levels of fumonisins were not classified as either B1 or B2 below the required regulatory limits (Table S6).

#### 4.3.3 Ochratoxins

Ochratoxins are produced mainly by *Aspergillus* and *Penicillium* species, including ochratoxins A, B, and C, although A is the most toxic and concern to human health [186]. Ochratoxin A causes kidney damage and negatively affects fetal development and the immune system. Ochratoxin A is a nephrotoxin that has also been implicated in kidney toxicity and cancer. Ochratoxin A has also been considered a liver toxin, an immune suppressant, a potent teratogen, and a carcinogen [186,195].

Ochratoxin A was measured in the five studies on ochratoxins (Table S6). The proportion of samples with mean ochratoxin A concentration exceeding the regulatory limit was 41.2% (14/34) (Table S6), a severe public health concern requiring stringent monitoring of HMPs by NRAs.

#### 4.3.4 Patulin

Patulin is a toxic chemical contaminant produced by *Aspergillus*, *Penicillium,* and *Byssochlamys* species [186]. It causes gastrointestinal, immunological, and neurological disorders [196]. Patulin is considered genotoxic, although its carcinogenic potential has not yet been demonstrated [186]. Patulin was measured in two of the reported studies (Table S6). The proportion of patulin within the required regulatory limits was 83.3% (5/6).

#### 4.3.5 Trichothecenes

Trichothecenes are sesquiterpenoid metabolites generated from several fungal genera [186]. Examples of trichothecenes are neosolaniol, diacetoxyscirpenol, fusarenon-x, nivalenol, and deoxynivalenol [186,197]. Health implications of trichothecenes in humans are gastrointestinal disorders and dermatitis [198].

Trichothecenes were measured in only two reported studies (Table 3). Specifically, diacetoxyscirpenol and deoxynivalenol were reported in one study each (Table S6). All the reported levels of deoxynivalenol (100%, 2/2) were within the specified regulatory limits (Table S6). However, the regulatory limits of diacetoxyscirpenol were not reported. Although trichothecene's health implications due to exposure are mild, resulting in nausea, vomiting, diarrhea, abdominal pain, headache, dizziness, and fever [199], their presence in HMPs requires stringent monitoring.

#### 4.3.6 Zearalenone

Zearalenone is a secondary estrogenic metabolite from *Fusarium* and *Gibberella* species, which enables it to bind to estrogen receptors of cells [186]. Consequently, a hormonal imbalance may occur that causes reproductive-related diseases such as prostate, ovarian, cervical, or breast cancers in humans [200]. Zearalenone was measured in two of the reported studies (Table S6). The proportion of zearalenone within the required regulatory limits is 80.0% (4/5).

### 4.4 Pesticides

Pesticides are chemical substances that control pests that injure cultivated plants or animals [201]. Pesticides may be classified as acaricides, algicide, avicides, bactericides, fungicides, herbicides, insecticides, molluscicides, nematicides, rodenticides, virucides, etc., based on their use, whiles their classification based on chemical composition includes organochlorines, organophosphorus, carbamates and pyrethrin and pyrethroids [23]. Direct and indirect exposure to pesticides and their consumption in food, water, fish, and herbal preparations may cause adverse health effects such as cancer, neurological effects, diabetes, respiratory diseases, fetal diseases, and human genetic disorders [[37], [38], [39], [40]].

Out of the five peer-reviewed articles, 100% (5/5), 40% (2/5), and 10% (1/5) were tested for organochlorine, organophosphate, and pyrethroid, respectively. Pesticides were measured only by studies from Africa and Asia. The only research from Africa that tested for pesticides focused on organochlorine, organophosphate, and pyrethroid [24]. However, the studies from Asia tested only organochlorine and organophosphate (Table 3).

#### 4.4.1 Organochlorine

Organochlorine pesticides are synthetic chlorinated hydrocarbon derivatives and persistent organic pollutants widely used to control pests. Examples include DDT, DDD, dicofol, aldrin, dieldrin, chlorobenziate, lindane, BHC, methoxychloro aldrin, chlordane, heptaclor, endosulfan, isodrin, isobenzan, toxaphene, and chloro propylate [23,202]. Due to their high toxicity, slow degradation, and bioaccumulation, short-term exposure may lead to gastrointestinal disorders, convulsions, headache, dizziness, tremors, confusion, muscle weakness, slurred speech, and sweating. Long-term exposure to organochlorine pesticides may damage the liver, kidney, central nervous system, thyroid, and bladder and cause cancer [202].

Twenty-seven different organochlorines were identified in 5 studies, which include 2,4-D, aldrin, chlordane cis, DDT, dieldrin, endosulfan alpha, endosulfan beta, endosulfan sulphate, endrin, HCH, HCH beta, HCH gamma, heptachlor, methoxychlor, total HCH, o, p’-DDT, p, p’-DDD, p, p’-DDE, p, p’-DDT, α-BHC, α-HCH, β-BHC, β-HCH, γ-HCH, γ-BHC, δ-BHC, and δ-HCH (Table S7).

The proportion of organochlorine contaminants, namely, 2,4-D, HCH, DDT, and total HCH that exceeded the required regulatory limits were 100% (1/1), 100% (20/20), 90.5% (19/21) and 66.7% (6/9), respectively (Table S7). Consequently, NRAs are expected to monitor organochlorine contaminants because of the potentially severe health implications. On the other hand, the reported levels of aldrin, chlordane cis, dieldrin, endosulfan alpha, endosulfan beta, endosulfan sulphate, endrin, HCH beta, HCH gamma, heptachlor, methoxychlor, o, p’-DDT, p, p’-DDD, p, p’-DDE, p, p’-DDT, α-BHC, α-HCH, β-BHC, β-HCH, γ-HCH, γ-BHC, δ-BHC, and δ-HCH were below the regulatory requirements (Table S7).

#### 4.4.2 Organophosphate

Organophosphate pesticides are chemical substances that are esters of phosphoric acid [202]. The widely used organophosphate pesticides include parathion, chlorpyrifos, diazinon, dichlorvos, phosmet, fenitrothion, tetrachlorvinphos, azamethiphos, azinphos-methyl, malathion, and methyl parathion [203]. Short-term exposure to organophosphate pesticides may lead to headache, dizziness, weakness, gastrointestinal disorders, blurred vision, slow pulse, difficulty breathing, coma, and neuropathy [204]. Long-term exposure to organophosphates leads to mainly neuropsychological disorders [205].

Twelve organophosphates were reported in two studies. The reported organophosphates were chlorfenvinphos, chlorpyrifos, diazinon, dimethoate, ethoprophos, fenitrothion, fonofos, malathion, methamidophos, parathion ethyl, pirimiphos methyl, and profenofos (Table S7).

None of the samples with reported mean concentration and regulatory limits of organophosphate and pyrethroid had their concentration above the regulatory limits (Table S7).

#### 4.4.3 Pyrethroids

Pyrethroids are synthetic organic derivatives of natural pyrethrins produced from the *Chrysanthemum cinerariaefolium* and *C. coccineum* [206]. Commonly used pyrethroids are allethrin, bonthrin, dimethrin, tetramethrin, ptrethrin, cyclethrin, furethrin, fenevelerate, alphamethrin, decamethrin, and cypermethrin [207]. Short-term exposure of humans to pyrethroids may cause paraesthesiae, and respiratory, eye, and skin irritation, whiles long-term exposure may have adverse effects on the reproductive and nervous systems [207].

Pyrethroids were reported in only one study, and the pyrethroids that were measured included bifenthrin, cyfluthrin I, cyfluthrin II, cyfluthrin III, cyfluthrin IV, cyhalothrin lambda, cypermethrin I, cypermethrin II, cypermethrin III, cypermethrin IV, deltamethrin, fenpropathrin, fenvalerate I, fenvalerate II, permethrin cis, and permethrin trans (Table S7).

Only one of the samples with reported mean concentration and regulatory limits of organophosphate and pyrethroid had their concentration above the regulatory limits (Table S7).

### 4.5 Residual solvents

Residual solvents are “volatile organic chemicals that are used or produced in the manufacture of drug substances or excipients or the preparation of medicinal products including herbal drug substances and herbal medicines” [22]. Depending on their adverse effects on human health and/or the environment, residual solvents have been classified as either class 1, class 2, or class 3 [208]. Residual solvents have been described as carcinogenic, environmentally hazardous, neurotoxic, and teratogenic in human health [41].

Four studies from Africa and Asia measured residual solvents, which were either class 1, class 2, class 3, or not classified (Table 3). Among the four studies, 50% (2/4), 100% (4/4), 100% (4/4), and 50% (2/4) tested for class 1, class 2, class 3, and unclassified residual solvents, respectively.

#### 4.5.1 Class 1

Class 1 residual solvents are organic solvents that are either known or strongly suspected human carcinogens [208]. Consequently, it should be avoided for the manufacture of orthodox and herbal medicinal products. However, where it is necessary to use such organic solvents to manufacture any medicinal product, their levels in the finished product must be controlled to the acceptable regulatory limits. Organic solvents classified as class 1 include 1,1,1-trichloroethane, 1,1-dichloroethene, 1,2-dichloroethane, benzene, and carbon tetrachloride [208].

All five different class 1 residual solvents were identified in the two studies reported on class 1 residual solvents. Carbon tetrachloride and benzene were identified in all two studies, while 1,1,1-trichloroethane, 1,1-dichloroethene, and 1,2-dichloroethane were identified in only one of the studies each (Table S8). Benzene (25.0%, 3/12) was the only class 1 residual solvent that exceeded the regulatory limit (Table S8). Therefore, NRAs are supposed to stringently monitor class 1 residual solvents because of their serious public health implications.

#### 4.5.2 Class 2

Class 2 residual solvents are organic, which are nongenotoxic animal carcinogens or may cause neurotoxicity, teratogenicity, and other reversible toxicities. Consequently, it should be limited to manufacture orthodox and herbal medicinal products, and their levels in the finished products must be within acceptable regulatory limits. Organic solvents classified as class 2 include 1,2-dimethoxyethane, 1,4-dioxane, 2,3-pentadione, acetonitrile, chlorobenzene, chloroform, *cis*-1,2-dichloroethene, cumene, cyclohexane, dichloromethane, ethylbenzene, hexane, methanol, methylbutylketone, methylcyclohexane, methylene chloride, nitromethane, pyridine, tetrahydrofuran, tetralin, toluene, *trans*-1,2-dichloroethene, trichloroethylene, xylene, m-xylene & p-xylene, and o-xylene [208].

All the known twenty-seven class 2 residual solvents were reported in the four studies that reported on class 2 residual solvents (Table S8). Acetonitrile, hexane, methanol, and toluene were the top class 2 residual solvents identified as they were identified in 3 out of the four studies that reported on class 2 residual solvents. Chloroform (14.3%, 1/7) was the only Class 2 residual solvent that exceeded the regulatory limit in the samples tested for chloroform (Table S8). Consequently, NRAs should monitor the levels of class 2 residual solvents because of their serious public health implications.

#### 4.5.3 Class 3

Class 3 residual solvents are organic with low toxic potential to humans and no required health-based exposure limit compared to class 1 and 2 residual solvents. Technically, it includes any organic solvent other than Class 1 and 2 residual solvents.

Twelve class 3 residual solvents were identified in the four studies. The identified class 3 residual solvents were 1-butanol, 1-propanol, 2-butanol, 2-propanol, acetic acid, acetone, ethanol, ethyl acetate, ethyl ether, isoamyl alcohol, isobutanol, and n-butyl acetate. The commonly identified class 3 residual solvents were acetone (75%, 3/4), ethanol (75%, 3/4), and ethyl acetate (75%, 3/4) (Table S8). Apart from ethanol (10.8%, 4/37), none of the other class 3 residual solvents had mean concentration levels exceeding the regulatory limits in the samples tested (Table S8).

#### 4.5.4 Not classified

The non-classified residual solvents can be considered technically Class 3 residual solvents, but they have not been specifically listed as such. The number of non-classified residual solvents was 7 (1-hexanol, acetoin, diacetyl, furfural, isoamyl acetate, octane, styrene) in the two papers that reported on residual solvents (Table S8). None of the non-classified residual solvents had mean concentration levels exceeding the regulatory limits in the samples tested (Table S8).

Some strategies have, however, been reported to reduce the contamination of HMPs. First, promoting good agricultural and collection practices during the growing, harvesting, and collection of medicinal plants/herbs can help minimize contamination of HMPs by ensuring that the starting materials are appropriately cultivated, harvested, and collected [209]. Second, properly storing the starting materials of HMPs can help prevent contamination since appropriate storage conditions, such as dry, cool, and well-ventilated areas, minimize the growth of bacteria and fungi [210].

Third, implementing quality control measures, such as testing for heavy metals, pesticides, residual solvents, mycotoxins, and microbial load, can help ensure that HMPs are of the required purity, safety, and efficacy standards [61]. To help ensure strict compliance with the above strategies, having appropriate regulatory frameworks can minimize HMP contamination and ensure product quality [42].

## 5 Analytical methods

Analytical methods that were used for the analysis of any of the contaminants (metals, microbial, mycotoxins, pesticides, residual solvents) were atomic absorption spectroscopy (AAS), fluorescent aptasensor pico green-based strategy (FAPS), gas chromatography (GC), gas chromatography mass spectrometry (GC-MS), inductively coupled plasma mass spectrometry (ICP-MS), inductively coupled plasma spectroscopy (ICPS), high-performance liquid chromatography (HPLC), atomic fluorescence spectrometry (AFS), high-performance liquid chromatography mass spectrometry (HPLC-MS), instrumental neutron activation analysis (INAA), thin-layer chromatography (TLC), and ultra-performance liquid chromatography mass spectrometry (UPLC-MS), which is consistent with published literature [211]. The determination of metal contaminants was measured using mainly AAS (51%, 26/51), ICPS (23.5%, 12/51), and ICP-MS (19.6%, 10/51) (Table S9). Analytical methods were mainly used to analyze microbial contamination, and mycotoxins were microbial culture and HPLC, respectively, while GC was mainly used for pesticides and residual solvents.

Validated analytical methods with high sensitivity are required to detect and quantify chemical contaminants in HMPs since their regulatory limits are generally in the ppm or ppb [61]. Chromatographic and various detection techniques are the most widely used analytical methods for contaminant determination [[211], [212], [213], [214]].

## 6 Strengths and limitations

This review acknowledges the following limitations: first, the literature search was limited to studies published in English, making it possible that some potentially relevant related publications may have been missed. Also, for some contaminants, such as organochlorine and residual solvents, the number of published peer-reviewed articles could have been higher, limiting generalizations of findings.

However, this is a crucial knowledge gap that our review has highlighted to guide future research and policy interventions. Another major strength of our study is the provision of an extensive report on the regional distribution, types, levels, and health risks of contaminants in HMPs, which is relevant for regulatory decisions.

## 7 Conclusion

While metals, microbial, mycotoxins, pesticides, and residual solvents were the reported contaminants in the 91 articles, metals, microbial, and mycotoxins were the most predominant. About 16% of the samples had their contaminant levels above regulatory limits, among which microbial contaminants had the highest proportion.

The presence of microbial and chemical contaminants in HMPs, notably more than regulatory limits, reflects the potential risk of severe microbial infections and other adverse health effects. Therefore, stricter regulatory interventions are needed to address the gaps in safe handling throughout the production chain, such as maintaining a clean environment and hygiene practices. Further studies to investigate the risk to consumers using appropriate risk assessment models are recommended to understand better the magnitude of the public health impact of microbial and chemical contamination of HMPs in LMICs.

## Data availability statement

Data included in article/supp. material/referenced in article.

## Author Contribution statement

Kwabena F.M. Opuni: Conceptualization; methodology; formal analysis; writing-original draft. James-Paul Kretchy: Formal analysis; writing-original draft. Joseph A. Boadi: Formal analysis; writing-review and editing. Kofi Agyabeng: Statistical analysis; writing-original draft. Theodosia Adanu: Methodology; data curation; Writing-review and editing. Samuel Ankamah: Methodology; data curation; writing-review and editing. Alexander Appiah: methodology; formal analysis; writing-review and editing. Geralda B. Amoah: methodology; formal analysis; writing-review and editing. Mariam Baidoo: methodology; formal analysis; writing-review and editing. Irene A. Kretchy: Conceptualization; methodology; writing-review and editing.

## Declaration of competing interest

The authors declare that they have no known competing financial interests or personal relationships that could have appeared to influence the work reported in this paper.
