# Supplementary material for: Contamination of herbal medicinal products in low-and-middle-income countries: A systematic review
Source: Heliyon. 2023 Aug 25;9(9):e19370. doi: 10.1016/j.heliyon.2023.e19370 (PMC10477504; doi:10.1016/j.heliyon.2023.e19370)
Supplement: Multimedia component 1 [file mmc1.docx]

**Supplementary data**

**Contamination of herbal medicinal products in low-and-middle-income countries: A systematic review**

Kwabena F. M. Opuni ^a, *^, James-Paul Kretchy ^b^, Kofi Agyabeng ^c^, Joseph A. Boadu ^a^, Theodosia Adanu ^d^, Samuel Ankamah ^d^, Alexander Appiah ^a^, Geralda B. Amoah ^a^, Mariam Baidoo ^a^, Irene A. Kretchy ^e^

^a^ Department of Pharmaceutical Chemistry, School of Pharmacy, University of Ghana, P.O. Box LG43, Legon, Accra, Ghana

^b^ Department of Public Health, School of Medicine and Health Sciences, Central University, P. O. Box 2305, Miotso, Accra, Ghana

^c^ Department of Biostatistics, School of Public Health, University of Ghana, P. O. Box LG13, Legon, Accra, Ghana

^d^ Balme Library, University of Ghana, P.O. Box LG24, Legon, Accra, Ghana

^e^ Department of Pharmacy Practice and Clinical Pharmacy, School of Pharmacy, University of Ghana, P.O. Box LG43, Legon, Accra, Ghana

* Corresponding Author.

Email: [kfopuni@ug.edu.gh](mailto:kfopuni@ug.edu.gh) (Kwabena F.M. Opuni)

**Supplementary tables**

**Table S1.** Search strategy

| Database | Search string | Citation | Date/Time |
| --- | --- | --- | --- |
| AJOL | (“low-and-middle-income countries” OR “low income countries” OR “middle income countries” OR “developing countries” OR “emerging countries” OR “underdeveloped countries” OR “poor countries” OR LMIC OR “low gross national” OR “low gross domestic”) AND (“herbal medicinal products” OR “herbal medicines” OR “traditional medicines” OR “indigenous medicines” OR herbs OR “natural health drugs” OR “medicinal drugs” OR “herbal drugs” OR “herbal preparations” OR “herbal products” OR “natural health products” OR “natural health preparations” OR “natural health drugs” OR HMP) AND (contamination OR “microbial load” OR “microbial contamination” OR “microbial adulteration” OR “microbial impurities” OR “bacterial load” OR “bacterial contamination” OR “bacterial adulteration” OR “bacterial impurities” OR “fungal load” OR “fungal contamination” OR “fungal adulteration” OR “fungal impurities” OR “protozoal load” OR “protozoal contamination” OR “protozoal adulteration” OR “protozoal impurities” OR “viral load” OR “viral contamination” OR “viral adulteration” OR “viral impurities” OR “spore contamination” OR “spore adulteration” OR “spore impurities” OR “chemical contamination” OR “chemical adulteration” OR “chemical impurities” OR mycotoxin OR “mycotoxin contamination” OR “mycotoxin adulteration” OR “mycotoxin impurities” OR “residual solvent” OR “residual solvent contamination” OR “residual solvent adulteration” OR “residual solvent impurities” OR “organic solvents” OR “organic solvents contamination” OR “organic solvents adulteration” OR “organic solvents impurities” OR “pesticide residues” OR “pesticide residues contamination” OR “pesticide residues adulteration” OR “pesticide residues impurities” OR “heavy metal” OR “heavy metal contamination” OR “heavy metal adulteration” OR “heavy metal impurities” OR “radioactive” OR “radioactive contamination” OR “radioactive adulteration” OR “radioactive impurities”) | 10,300*  *77 were the results the database displayed for retrieval. | 10:41 AM  15/04/2021  To  12:48 PM  17/04/2021 |
|  | contamination OR “microbial load” OR “microbial contamination” OR “microbial adulteration” OR “microbial impurities” OR “bacterial load” OR “bacterial contamination” OR “bacterial adulteration” OR “bacterial impurities” OR “fungal load” OR “fungal contamination” OR “fungal adulteration” OR “fungal impurities” OR “protozoal load” OR “protozoal contamination” OR “protozoal adulteration” OR “protozoal impurities” OR “viral load” OR “viral contamination” OR “viral adulteration” OR “viral impurities” OR “spore contamination” OR “spore adulteration” OR “spore impurities” OR “chemical contamination” OR “chemical adulteration” OR “chemical impurities” OR mycotoxin OR “mycotoxin contamination” OR “mycotoxin adulteration” OR “mycotoxin impurities” OR “residual solvent” OR “residual solvent contamination” OR “residual solvent adulteration” OR “residual solvent impurities” OR “organic solvents” OR “organic solvents contamination” OR “organic solvents adulteration” OR “organic solvents impurities” OR “pesticide residues” OR “pesticide residues contamination” OR “pesticide residues adulteration” OR “pesticide residues impurities” OR “heavy metal” OR “heavy metal contamination” OR “heavy metal adulteration” OR “heavy metal impurities” OR “radioactive” OR “radioactive contamination” OR “radioactive adulteration” OR “radioactive impurities” | 16,800 | 10:40 AM  15/04/2021 |
|  | “herbal medicinal products” OR “herbal medicines” OR “traditional medicines” OR “indigenous medicines” OR herbs OR “natural health drugs” OR “medicinal drugs” OR “herbal drugs” OR “herbal preparations” OR “herbal products” OR “natural health products” OR “natural health preparations” OR “natural health drugs” OR HMP | 11,300 | 10:39 AM  15/04/2021 |
|  | “low-and-middle-income countries” OR “low income countries” OR “middle income countries” OR “developing countries” OR “emerging countries” OR “underdeveloped countries” OR “poor countries” OR LMIC OR “low gross national” OR “low gross domestic” | 45,000 | 10:38 AM  15/04/2021 |
|  |  |  |  |
| DOAJ | “low-and-middle-income countries” OR “low income countries” OR “middle income countries” OR “developing countries” OR “emerging countries” OR “underdeveloped countries” OR “poor countries” OR LMIC OR “low gross national” OR “low gross domestic” | 83 | 10:33 AM  15/04/2021 |
|  | “herbal medicinal products” OR “herbal medicines” OR “traditional medicines” OR “indigenous medicines” OR herbs OR “natural health drugs” OR “medicinal drugs” OR “herbal drugs” OR “herbal preparations” OR “herbal products” OR “natural health products” OR “natural health preparations” OR “natural health drugs” OR HMP | 763 | 10:33 AM  15/04/2021 |
|  | contamination OR “microbial load” OR “microbial contamination” OR “microbial adulteration” OR “microbial impurities” OR “bacterial load” OR “bacterial contamination” OR “bacterial adulteration” OR “bacterial impurities” OR “fungal load” OR “fungal contamination” OR “fungal adulteration” OR “fungal impurities” OR “protozoal load” OR “protozoal contamination” OR “protozoal adulteration” OR “protozoal impurities” OR “viral load” OR “viral contamination” OR “viral adulteration” OR “viral impurities” OR “spore contamination” OR “spore adulteration” OR “spore impurities” OR “chemical contamination” OR “chemical adulteration” OR “chemical impurities” OR mycotoxin OR “mycotoxin contamination” OR “mycotoxin adulteration” OR “mycotoxin impurities” OR “residual solvent” OR “residual solvent contamination” OR “residual solvent adulteration” OR “residual solvent impurities” OR “organic solvents” OR “organic solvents contamination” OR “organic solvents adulteration” OR “organic solvents impurities” OR “pesticide residues” OR “pesticide residues contamination” OR “pesticide residues adulteration” OR “pesticide residues impurities” OR “heavy metal” OR “heavy metal contamination” OR “heavy metal adulteration” OR “heavy metal impurities” OR “radioactive” OR “radioactive contamination” OR “radioactive adulteration” OR “radioactive impurities” | 0 | 10:34 AM  15/04/2021 |
|  |  |  |  |
| HINARI | “low-and-middle-income countries” OR “low income countries” OR “middle income countries” OR “developing countries” OR “emerging countries” OR “underdeveloped countries” OR “poor countries” OR LMIC OR “low gross national” OR “low gross domestic” | 537,007 | 01:45 PM  14/04/2021 |
|  | “herbal medicinal products” OR “herbal medicines” OR “traditional medicines” OR “indigenous medicines” OR herbs OR “natural health drugs” OR “medicinal drugs” OR “herbal drugs” OR “herbal preparations” OR “herbal products” OR “natural health products” OR “natural health preparations” OR “natural health drugs” OR HMP | 240,906 | 01:46 PM  14/04/2021 |
|  | contamination OR “microbial load” OR “microbial contamination” OR “microbial adulteration” OR “microbial impurities” OR “bacterial load” OR “bacterial contamination” OR “bacterial adulteration” OR “bacterial impurities” OR “fungal load” OR “fungal contamination” OR “fungal adulteration” OR “fungal impurities” OR “protozoal load” OR “protozoal contamination” OR “protozoal adulteration” OR “protozoal impurities” OR “viral load” OR “viral contamination” OR “viral adulteration” OR “viral impurities” OR “spore contamination” OR “spore adulteration” OR “spore impurities” OR “chemical contamination” OR “chemical adulteration” OR “chemical impurities” OR mycotoxin OR “mycotoxin contamination” OR “mycotoxin adulteration” OR “mycotoxin impurities” OR “residual solvent” OR “residual solvent contamination” OR “residual solvent adulteration” OR “residual solvent impurities” OR “organic solvents” OR “organic solvents contamination” OR “organic solvents adulteration” OR “organic solvents impurities” OR “pesticide residues” OR “pesticide residues contamination” OR “pesticide residues adulteration” OR “pesticide residues impurities” OR “heavy metal” OR “heavy metal contamination” OR “heavy metal adulteration” OR “heavy metal impurities” OR “radioactive” OR “radioactive contamination” OR “radioactive adulteration” OR “radioactive impurities” | 1,331,401 | 01:47 PM  14/04/2021 |
|  | (“low-and-middle-income countries” OR “low income countries” OR “middle income countries” OR “developing countries” OR “emerging countries” OR “underdeveloped countries” OR “poor countries” OR LMIC OR “low gross national” OR “low gross domestic”) AND (“herbal medicinal products” OR “herbal medicines” OR “traditional medicines” OR “indigenous medicines” OR herbs OR “natural health drugs” OR “medicinal drugs” OR “herbal drugs” OR “herbal preparations” OR “herbal products” OR “natural health products” OR “natural health preparations” OR “natural health drugs” OR HMP) AND (contamination OR “microbial load” OR “microbial contamination” OR “microbial adulteration” OR “microbial impurities” OR “bacterial load” OR “bacterial contamination” OR “bacterial adulteration” OR “bacterial impurities” OR “fungal load” OR “fungal contamination” OR “fungal adulteration” OR “fungal impurities” OR “protozoal load” OR “protozoal contamination” OR “protozoal adulteration” OR “protozoal impurities” OR “viral load” OR “viral contamination” OR “viral adulteration” OR “viral impurities” OR “spore contamination” OR “spore adulteration” OR “spore impurities” OR “chemical contamination” OR “chemical adulteration” OR “chemical impurities” OR mycotoxin OR “mycotoxin contamination” OR “mycotoxin adulteration” OR “mycotoxin impurities” OR “residual solvent” OR “residual solvent contamination” OR “residual solvent adulteration” OR “residual solvent impurities” OR “organic solvents” OR “organic solvents contamination” OR “organic solvents adulteration” OR “organic solvents impurities” OR “pesticide residues” OR “pesticide residues contamination” OR “pesticide residues adulteration” OR “pesticide residues impurities” OR “heavy metal” OR “heavy metal contamination” OR “heavy metal adulteration” OR “heavy metal impurities” OR “radioactive” OR “radioactive contamination” OR “radioactive adulteration” OR “radioactive impurities”) | 487*  *About 2,953 results for search query. However, the available results were 487. | 10:11 AM  15/04/2021 |
|  |  |  |  |
| CINAHL | S4 (S1 AND S2 AND S3) | 3 | 9:37:25 AM  14/4/2021 |
|  | S3  contamination OR “microbial load” OR “microbial contamination” OR “microbial adulteration” OR “microbial impurities” OR “bacterial load” OR “bacterial contamination” OR “bacterial adulteration” OR “bacterial impurities” OR “fungal load” OR “fungal contamination” OR “fungal adulteration” OR “fungal impurities” OR “protozoal load” OR “protozoal contamination” OR “protozoal adulteration” OR “protozoal impurities” OR “viral load” OR “viral contamination” OR “viral adulteration” OR “viral impurities” OR “spore contamination” OR “spore adulteration” OR “spore impurities” OR “chemical contamination” OR “chemical adulteration” OR “chemical impurities” OR mycotoxin OR “mycotoxin contamination” OR “mycotoxin adulteration” OR “mycotoxin impurities” OR “residual solvent” OR “residual solvent contamination” OR “residual solvent adulteration” OR “residual solvent impurities” OR “organic solvents” OR “organic solvents contamination” OR “organic solvents adulteration” OR “organic solvents impurities” OR “pesticide residues” OR “pesticide residues contamination” OR “pesticide residues adulteration” OR “pesticide residues impurities” OR “heavy metal” OR “heavy metal contamination” OR “heavy metal adulteration” OR “heavy metal impurities” OR “radioactive” OR “radioactive contamination” OR “radioactive adulteration” OR “radioactive impurities” | 39,381 |  |
|  | S2  “herbal medicinal products” OR “herbal medicines” OR “traditional medicines” OR “indigenous medicines” OR herbs OR “natural health drugs” OR “medicinal drugs” OR “herbal drugs” OR “herbal preparations” OR “herbal products” OR “natural health products” OR “natural health preparations” OR “natural  health drugs” OR HMP | 17,183 |  |
|  | S1  “low-and-middle-income countries” OR “low income countries” OR “middle income countries” OR “developing countries” OR “emerging countries” OR “underdeveloped countries” OR “poor countries” OR LMIC OR “low gross national” OR “low gross domestic” | 37,533 |  |
|  |  |  |  |
| PubMed | S4  ((#1) AND (#2)) AND (#3) | 45 | 08:57:41  14/04/2021 |
|  | S3  "contaminant"[All Fields] OR "contaminant s"[All Fields] OR "contaminants"[All Fields] OR "contaminate"[All Fields] OR "contaminated"[All Fields] OR "contaminates"[All Fields] OR "contaminating"[All Fields] OR "contamination"[All Fields] OR "contaminations"[All Fields] OR "contaminative"[All Fields] OR "contamined"[All Fields] OR "microbial load"[All Fields] OR "microbial contamination"[All Fields] OR (("microbial"[All Fields] OR "microbially"[All Fields] OR "microbials"[All Fields]) AND ("adulterant"[All Fields] OR "adulterants"[All Fields] OR "adulterate"[All Fields] OR "adulterated"[All Fields] OR "adulterates"[All Fields] OR "adulterating"[All Fields] OR "adulteration"[All Fields] OR "adulterations"[All Fields])) OR (("microbial"[All Fields] OR "microbially"[All Fields] OR "microbials"[All Fields]) AND ("impure"[All Fields] OR "impurities"[All Fields] OR "impurity"[All Fields])) OR "bacterial load"[All Fields] OR "bacterial contamination"[All Fields] OR (("bacterial"[All Fields] OR "bacterially"[All Fields] OR "bacterials"[All Fields]) AND ("adulterant"[All Fields] OR "adulterants"[All Fields] OR "adulterate"[All Fields] OR "adulterated"[All Fields] OR "adulterates"[All Fields] OR "adulterating"[All Fields] OR "adulteration"[All Fields] OR "adulterations"[All Fields])) OR "bacterial impurities"[All Fields] OR "fungal load"[All Fields] OR "fungal contamination"[All Fields] OR (("fungals"[All Fields] OR "microbiology"[MeSH Terms] OR "microbiology"[All Fields] OR "fungal"[All Fields] OR "fungi"[MeSH Terms] OR "fungi"[All Fields]) AND ("adulterant"[All Fields] OR "adulterants"[All Fields] OR "adulterate"[All Fields] OR "adulterated"[All Fields] OR "adulterates"[All Fields] OR "adulterating"[All Fields] OR "adulteration"[All Fields] OR "adulterations"[All Fields])) OR (("fungals"[All Fields] OR "microbiology"[MeSH Terms] OR "microbiology"[All Fields] OR "fungal"[All Fields] OR "fungi"[MeSH Terms] OR "fungi"[All Fields]) AND ("impure"[All Fields] OR "impurities"[All Fields] OR "impurity"[All Fields])) OR ("protozoal"[All Fields] AND "load"[All Fields]) OR "protozoal contamination"[All Fields] OR ("protozoal"[All Fields] AND ("adulterant"[All Fields] OR "adulterants"[All Fields] OR "adulterate"[All Fields] OR "adulterated"[All Fields] OR "adulterates"[All Fields] OR "adulterating"[All Fields] OR "adulteration"[All Fields] OR "adulterations"[All Fields])) OR ("protozoal"[All Fields] AND ("impure"[All Fields] OR "impurities"[All Fields] OR "impurity"[All Fields])) OR "viral load"[All Fields] OR "viral contamination"[All Fields] OR (("virally"[All Fields] OR "virals"[All Fields] OR "virology"[MeSH Terms] OR "virology"[All Fields] OR "viral"[All Fields]) AND ("adulterant"[All Fields] OR "adulterants"[All Fields] OR "adulterate"[All Fields] OR "adulterated"[All Fields] OR "adulterates"[All Fields] OR "adulterating"[All Fields] OR "adulteration"[All Fields] OR "adulterations"[All Fields])) OR "viral impurities"[All Fields] OR "spore contamination"[All Fields] OR (("spore s"[All Fields] OR "spored"[All Fields] OR "spores"[MeSH Terms] OR "spores"[All Fields] OR "spore"[All Fields] OR "sporing"[All Fields]) AND ("adulterant"[All Fields] OR "adulterants"[All Fields] OR "adulterate"[All Fields] OR "adulterated"[All Fields] OR "adulterates"[All Fields] OR "adulterating"[All Fields] OR "adulteration"[All Fields] OR "adulterations"[All Fields])) OR (("spore s"[All Fields] OR "spored"[All Fields] OR "spores"[MeSH Terms] OR "spores"[All Fields] OR "spore"[All Fields] OR "sporing"[All Fields]) AND ("impure"[All Fields] OR "impurities"[All Fields] OR "impurity"[All Fields])) OR "chemical contamination"[All Fields] OR "chemical adulteration"[All Fields] OR "chemical impurities"[All Fields] OR ("mycotoxines"[All Fields] OR "mycotoxins"[MeSH Terms] OR "mycotoxins"[All Fields] OR "mycotoxin"[All Fields]) OR "mycotoxin contamination"[All Fields] OR (("mycotoxines"[All Fields] OR "mycotoxins"[MeSH Terms] OR "mycotoxins"[All Fields] OR "mycotoxin"[All Fields]) AND ("adulterant"[All Fields] OR "adulterants"[All Fields] OR "adulterate"[All Fields] OR "adulterated"[All Fields] OR "adulterates"[All Fields] OR "adulterating"[All Fields] OR "adulteration"[All Fields] OR "adulterations"[All Fields])) OR (("mycotoxines"[All Fields] OR "mycotoxins"[MeSH Terms] OR "mycotoxins"[All Fields] OR "mycotoxin"[All Fields]) AND ("impure"[All Fields] OR "impurities"[All Fields] OR "impurity"[All Fields])) OR "residual solvent"[All Fields] OR (("residual"[All Fields] OR "residuals"[All Fields]) AND ("solvent s"[All Fields] OR "solvents"[Pharmacological Action] OR "solvents"[MeSH Terms] OR "solvents"[All Fields] OR "solvent"[All Fields]) AND ("contaminant"[All Fields] OR "contaminant s"[All Fields] OR "contaminants"[All Fields] OR "contaminate"[All Fields] OR "contaminated"[All Fields] OR "contaminates"[All Fields] OR "contaminating"[All Fields] OR "contamination"[All Fields] OR "contaminations"[All Fields] OR "contaminative"[All Fields] OR "contamined"[All Fields])) OR (("residual"[All Fields] OR "residuals"[All Fields]) AND ("solvent s"[All Fields] OR "solvents"[Pharmacological Action] OR "solvents"[MeSH Terms] OR "solvents"[All Fields] OR "solvent"[All Fields]) AND ("adulterant"[All Fields] OR "adulterants"[All Fields] OR "adulterate"[All Fields] OR "adulterated"[All Fields] OR "adulterates"[All Fields] OR "adulterating"[All Fields] OR "adulteration"[All Fields] OR "adulterations"[All Fields])) OR (("residual"[All Fields] OR "residuals"[All Fields]) AND ("solvent s"[All Fields] OR "solvents"[Pharmacological Action] OR "solvents"[MeSH Terms] OR "solvents"[All Fields] OR "solvent"[All Fields]) AND ("impure"[All Fields] OR "impurities"[All Fields] OR "impurity"[All Fields])) OR "organic solvents"[All Fields] OR (("organic"[All Fields] OR "organically"[All Fields] OR "organics"[All Fields]) AND ("solvent s"[All Fields] OR "solvents"[Pharmacological Action] OR "solvents"[MeSH Terms] OR "solvents"[All Fields] OR "solvent"[All Fields]) AND ("contaminant"[All Fields] OR "contaminant s"[All Fields] OR "contaminants"[All Fields] OR "contaminate"[All Fields] OR "contaminated"[All Fields] OR "contaminates"[All Fields] OR "contaminating"[All Fields] OR "contamination"[All Fields] OR "contaminations"[All Fields] OR "contaminative"[All Fields] OR "contamined"[All Fields])) OR (("organic"[All Fields] OR "organically"[All Fields] OR "organics"[All Fields]) AND ("solvent s"[All Fields] OR "solvents"[Pharmacological Action] OR "solvents"[MeSH Terms] OR "solvents"[All Fields] OR "solvent"[All Fields]) AND ("adulterant"[All Fields] OR "adulterants"[All Fields] OR "adulterate"[All Fields] OR "adulterated"[All Fields] OR "adulterates"[All Fields] OR "adulterating"[All Fields] OR "adulteration"[All Fields] OR "adulterations"[All Fields])) OR (("organic"[All Fields] OR "organically"[All Fields] OR "organics"[All Fields]) AND ("solvent s"[All Fields] OR "solvents"[Pharmacological Action] OR "solvents"[MeSH Terms] OR "solvents"[All Fields] OR "solvent"[All Fields]) AND ("impure"[All Fields] OR "impurities"[All Fields] OR "impurity"[All Fields])) OR "pesticide residues"[All Fields] OR (("pesticide residues"[MeSH Terms] OR ("pesticide"[All Fields] AND "residues"[All Fields]) OR "pesticide residues"[All Fields]) AND ("contaminant"[All Fields] OR "contaminant s"[All Fields] OR "contaminants"[All Fields] OR "contaminate"[All Fields] OR "contaminated"[All Fields] OR "contaminates"[All Fields] OR "contaminating"[All Fields] OR "contamination"[All Fields] OR "contaminations"[All Fields] OR "contaminative"[All Fields] OR "contamined"[All Fields])) OR (("pesticide residues"[MeSH Terms] OR ("pesticide"[All Fields] AND "residues"[All Fields]) OR "pesticide residues"[All Fields]) AND ("adulterant"[All Fields] OR "adulterants"[All Fields] OR "adulterate"[All Fields] OR "adulterated"[All Fields] OR "adulterates"[All Fields] OR "adulterating"[All Fields] OR "adulteration"[All Fields] OR "adulterations"[All Fields])) OR (("pesticide residues"[MeSH Terms] OR ("pesticide"[All Fields] AND "residues"[All Fields]) OR "pesticide residues"[All Fields]) AND ("impure"[All Fields] OR "impurities"[All Fields] OR "impurity"[All Fields])) OR "heavy metal"[All Fields] OR "heavy metal contamination"[All Fields] OR (("metals, heavy"[MeSH Terms] OR ("metals"[All Fields] AND "heavy"[All Fields]) OR "heavy metals"[All Fields] OR ("heavy"[All Fields] AND "metal"[All Fields]) OR "heavy metal"[All Fields]) AND ("adulterant"[All Fields] OR "adulterants"[All Fields] OR "adulterate"[All Fields] OR "adulterated"[All Fields] OR "adulterates"[All Fields] OR "adulterating"[All Fields] OR "adulteration"[All Fields] OR "adulterations"[All Fields])) OR "heavy metal impurities"[All Fields] OR "radioactive"[All Fields] OR "radioactive contamination"[All Fields] OR (("radioactively"[All Fields] OR "radioactivity"[MeSH Terms] OR "radioactivity"[All Fields] OR "radioactive"[All Fields] OR "radioactivities"[All Fields]) AND ("adulterant"[All Fields] OR "adulterants"[All Fields] OR "adulterate"[All Fields] OR "adulterated"[All Fields] OR "adulterates"[All Fields] OR "adulterating"[All Fields] OR "adulteration"[All Fields] OR "adulterations"[All Fields])) OR "radioactive impurities"[All Fields] | 515,177 | 08:47:28 |
|  | S2  "herbal medicinal products"[All Fields] OR "herbal medicines"[All Fields] OR "traditional medicines"[All Fields] OR "indigenous medicines"[All Fields] OR ("herb s"[All Fields] OR "herbes"[All Fields] OR "herbs"[All Fields]) OR (("natural"[All Fields] OR "naturally"[All Fields] OR "naturals"[All Fields] OR "nature"[MeSH Terms] OR "nature"[All Fields] OR "nature s"[All Fields] OR "natures"[All Fields]) AND ("health"[MeSH Terms] OR "health"[All Fields] OR "health s"[All Fields] OR "healthful"[All Fields] OR "healthfulness"[All Fields] OR "healths"[All Fields]) AND ("drug s"[All Fields] OR "pharmaceutical preparations"[MeSH Terms] OR ("pharmaceutical"[All Fields] AND "preparations"[All Fields]) OR "pharmaceutical preparations"[All Fields] OR "drugs"[All Fields])) OR "medicinal drugs"[All Fields] OR "herbal drugs"[All Fields] OR "herbal preparations"[All Fields] OR "herbal products"[All Fields] OR "natural health products"[All Fields] OR (("natural"[All Fields] OR "naturally"[All Fields] OR "naturals"[All Fields] OR "nature"[MeSH Terms] OR "nature"[All Fields] OR "nature s"[All Fields] OR "natures"[All Fields]) AND ("health"[MeSH Terms] OR "health"[All Fields] OR "health s"[All Fields] OR "healthful"[All Fields] OR "healthfulness"[All Fields] OR "healths"[All Fields]) AND ("preparate"[All Fields] OR "preparates"[All Fields] OR "preparation"[All Fields] OR "preparations"[All Fields] OR "preparative"[All Fields] OR "preparatively"[All Fields] OR "prepare"[All Fields] OR "prepared"[All Fields] OR "prepares"[All Fields] OR "preparing"[All Fields])) OR (("natural"[All Fields] OR "naturally"[All Fields] OR "naturals"[All Fields] OR "nature"[MeSH Terms] OR "nature"[All Fields] OR "nature s"[All Fields] OR "natures"[All Fields]) AND ("health"[MeSH Terms] OR "health"[All Fields] OR "health s"[All Fields] OR "healthful"[All Fields] OR "healthfulness"[All Fields] OR "healths"[All Fields]) AND ("drug s"[All Fields] OR "pharmaceutical preparations"[MeSH Terms] OR ("pharmaceutical"[All Fields] AND "preparations"[All Fields]) OR "pharmaceutical preparations"[All Fields] OR "drugs"[All Fields])) OR "HMP"[All Fields] | 95,085 | 08:41:46 |
|  | S1  "low-and-middle-income countries"[All Fields] OR "low income countries"[All Fields] OR "middle income countries"[All Fields] OR "developing countries"[All Fields] OR "emerging countries"[All Fields] OR "underdeveloped countries"[All Fields] OR "poor countries"[All Fields] OR "LMIC"[All Fields] OR "low gross national"[All Fields] OR "low gross domestic"[All Fields] | 149,049 | 08:41:01 |
|  |  |  |  |
| SCOPUS | ( ( TITLE-ABS-KEY ( "herbal medicinal product*" ) OR TITLE- ABS-KEY ( hmp ) OR TITLE-ABS-KEY ( "indegenous medicin*" ) OR TITLE-ABS-KEY ( "herbal medicin*" ) OR TITLE-ABS-KEY ( "herbal substance*" ) OR TITLE-ABS-KEY ( "herbal mi xtur*" ) OR TITLE-ABS-KEY ( "traditional medicin*" ) OR TITLE-ABS-KEY ( "natural medicin*" ) OR TITLE-ABS-KEY ( herb ) OR TITLE-ABS-KEY ( "natural health drug" ) OR TITLE-A BS-KEY ( "medicinal drug" ) OR TITLE-ABS-KEY ( "herbal drug" ) OR TITLE-ABS-KEY ( "herbal preparation" ) OR TITLE-ABS-KEY ( "herbal product" ) OR TITLE-ABS-KEY ( "natural he alth product" ) OR TITLE-ABS-KEY ( "natural health preparati on" ) OR TITLE-ABS-KEY ( "natural health drug" ) ) ) AND ( ( TITLE-ABS-KEY ( microbial ) OR TITLE-ABS-KEY ( cont amin* ) OR TITLE-ABS-KEY ( impurities ) OR TITLE-ABS-K EY ( adulteration* ) OR TITLE-ABS-KEY ( bacteri* ) OR TITL E-ABS-KEY ( fungal ) OR TITLE-ABS-KEY ( protozoal ) OR TITLE-ABS-KEY ( viral ) OR TITLE-ABS-KEY ( spore ) O R TITLE-ABS-KEY ( chemical ) OR TITLE-ABS-KEY ( mycotoxin ) OR TITLE-ABS-KEY ( "residual solvent" ) OR TITLE-ABS-KEY ( "pesticide residue" ) OR TITLE-ABS-KEY ( "heavy metal" ) OR TITLE-ABS-KEY ( radioactive ) OR TITLE-ABS- KEY ( mycotoxin ) OR TITLE-ABS-KEY ( "organic solvent" ) ) ) AND ( ( TITLE-ABS-KEY ( "low and middle income" ) OR TITLE-ABS-KEY ( "developing countries" ) OR TITLE-ABS- KEY ( "low-and-middle-income" ) OR TITLE-ABS-KEY ( africa ) OR TITLE-ABS-KEY ( "West Africa" ) OR TITLE-ABS-KE Y ( "third world" ) OR TITLE-ABS-KEY ( "low income countries" ) OR TITLE-ABS-KEY ( "middle income countries" ) OR TITLE-ABS-KEY ( "emerging countries" ) OR TITLE-ABS-KEY ( " under developped countries" ) OR TITLE-ABS-KEY ( lmic ) O R TITLE-ABS-KEY ( "poor countries" ) OR TITLE-ABS-KEY ( "low gross national" ) OR TITLE-ABS-KEY ( "low gross domestic" ) ) ) | 1,374 | 9/4/2021 |
|  |  |  |  |
| WHO GIM | “low-and-middle-income countries” OR “low income countries” OR “middle income countries” OR “developing countries” OR “emerging countries” OR “underdeveloped countries” OR “poor countries” OR LMIC OR “low gross national” OR “low gross domestic” | 12 | 01:21 PM  14/02/2021 |
|  | “herbal medicinal products” OR “herbal medicines” OR “traditional medicines” OR “indigenous medicines” OR herbs OR “natural health drugs” OR “medicinal drugs” OR “herbal drugs” OR “herbal preparations” OR “herbal products” OR “natural health products” OR “natural health preparations” OR “natural health drugs” OR HMP | 475 | 01:22 PM  14/02/2021 |
|  | contamination OR “microbial load” OR “microbial contamination” OR “microbial adulteration” OR “microbial impurities” OR “bacterial load” OR “bacterial contamination” OR “bacterial adulteration” OR “bacterial impurities” OR “fungal load” OR “fungal contamination” OR “fungal adulteration” OR “fungal impurities” OR “protozoal load” OR “protozoal contamination” OR “protozoal adulteration” OR “protozoal impurities” OR “viral load” OR “viral contamination” OR “viral adulteration” OR “viral impurities” OR “spore contamination” OR “spore adulteration” OR “spore impurities” OR “chemical contamination” OR “chemical adulteration” OR “chemical impurities” OR mycotoxin OR “mycotoxin contamination” OR “mycotoxin adulteration” OR “mycotoxin impurities” OR “residual solvent” OR “residual solvent contamination” OR “residual solvent adulteration” OR “residual solvent impurities” OR “organic solvents” OR “organic solvents contamination” OR “organic solvents adulteration” OR “organic solvents impurities” OR “pesticide residues” OR “pesticide residues contamination” OR “pesticide residues adulteration” OR “pesticide residues impurities” OR “heavy metal” OR “heavy metal contamination” OR “heavy metal adulteration” OR “heavy metal impurities” OR “radioactive” OR “radioactive contamination” OR “radioactive adulteration” OR “radioactive impurities” | 0 | 01:23 PM  14/02/2021 |

AJOL, Africa journal online; CINAHL, Cumulative Index to Nursing and Allied Health Literature; DOAJ, Directory of Open Access Journals; HINARI, Health Inter-Network Access to Research Initiative; WHO GIM, World Health Organization Global Index Medicus.

**Table S2**. Quality appraisal scoring system

| **Quality appraisal dimensions** | **Code** | **Points awarded** |
| --- | --- | --- |
| Country study was conducted | A | 1 |
| HMPs analysed |  |  |
| Name of HMP | B | 1 |
| HMP dosage / formulation form | C | 1 |
| Active components of the preparation | D | 1 |
| Source of samples (including location) | E | 1 |
| Analysis of HMP samples |  |  |
| Assay method used | F | 1 |
| Sampling method used | G | 1 |
| Total number of samples analysed | H | 1 |
| Analysis for contaminants |  |  |
| Type of contaminants in HMP | I | 1 |
| Number of contaminants tested | J | 1 |
| Number of contaminants detected and identified | K | 1 |
| Levels of contaminants | L | 1 |
| Factors associated with the observed contamination | M | 1 |
| Health outcome(s) assessed | N | 1 |
| **Total** |  | **14** |

**Table S3.** Quality of articles included in the study

|  | **Code ^a)^** | | | | | | | | | | | | | | **Total** | **Quality** | **Reference** |
| --- | --- | --- | --- | --- | --- | --- | --- | --- | --- | --- | --- | --- | --- | --- | --- | --- | --- |
|  | **A** | **B** | **C** | **D** | **E** | **F** | **G** | **H** | **I** | **J** | **K** | **L** | **M** | **N** |  |  |  |
| 1 | 1 | 1 | 0 | 1 | 0 | 1 | 0 | 1 | 1 | 1 | 1 | 1 | 1 | 1 | 11 | Good | [1] |
| 2 | 1 | - | 1 | 1 | 1 | 1 | 1 | - | 1 | 1 | 1 | 1 | 1 | 1 | 12 | Good | [2] |
| 3 | 1 | 1 | 1 | 0 | 1 | 1 | 0 | 1 | 1 | 1 | 1 | 1 | 1 | 1 | 12 | Good | [3] |
| 4 | 1 | 0 | 1 | 0 | 1 | 1 | 0 | 1 | 1 | 1 | 1 | 1 | 0 | 1 | 10 | Good | [4] |
| 5 | 1 | 0 | 1 | 1 | 1 | 1 | 0 | 1 | 1 | 1 | 1 | 1 | 0 | 0 | 10 | Good | [5] |
| 6 | 1 | 0 | 1 | 0 | 1 | 1 | 1 | 1 | 1 | 1 | 1 | 1 | 1 | 1 | 12 | Good | [6] |
| 7 | 1 | 1 | 1 | 1 | 1 | 1 | 0 | 1 | 1 | 0 | 0 | 1 | 1 | 1 | 11 | Good | [7] |
| 8 | 1 | 1 | 1 | - | 1 | 1 | 1 | - | 1 | 1 | 1 | 1 | 1 | 1 | 12 | Good | [8] |
| 9 | 1 | - | 1 | 1 | 1 | 1 | 1 | - | 1 | 1 | 1 | 1 | 1 | - | 11 | Good | [9] |
| 10 | 1 | 1 | 1 | 1 | 1 | 1 | 0 | 1 | 1 | 1 | 1 | 1 | 1 | 0 | 12 | Good | [10] |
| 11 | 1 | 1 | 1 | 0 | 0 | 1 | 0 | 0 | 1 | 1 | 1 | 1 | 0 | 0 | 8 | Fair | [11] |
| 12 | 1 | 0 | 1 | 0 | 1 | 1 | 1 | 1 | 1 | 1 | 1 | 0 | 1 | 0 | 10 | Good | [12] |
| 13 | 1 | - | - | 1 | 1 | 1 | 1 | 1 | 1 | 1 | 1 | 1 | 1 | 1 | 12 | Good | [13] |
| 14 | 1 | 0 | 0 | 1 | 1 | 1 | 0 | 1 | 1 | 1 | 1 | 1 | 1 | 0 | 10 | Good | [14] |
| 15 | 1 | 0 | 1 | 1 | 1 | 1 | 0 | 1 | 1 | 1 | 1 | 1 | 0 | 0 | 10 | Good | [15] |
| 16 | 1 | - | - | 1 | 1 | 1 | 1 | - | 1 | 1 | 1 | 1 | 1 | 1 | 11 | Good | [16] |
| 17 | 1 | 0 | 1 | 1 | 1 | 1 | 0 | 1 | 1 | 1 | 1 | 1 | 1 | 0 | 11 | Good | [17] |
| 18 | 1 | - | 1 | 1 | 1 | 1 | 1 | 1 | 1 | 1 | 1 | 1 | 1 | 1 | 13 | Good | [18] |
| 19 | 1 | 1 | 1 | 1 | 1 | 1 | 0 | 0 | 1 | 1 | 1 | 1 | 1 | 0 | 11 | Good | [19] |
| 20 | 1 | 0 | 1 | 1 | 1 | 1 | 1 | 1 | 1 | 1 | 1 | 1 | 1 | 0 | 12 | Good | [20] |
| 21 | 1 | - | - | 1 | 1 | 1 | 1 | 1 | 1 | 1 | 1 | 1 | 1 | 1 | 12 | Good | [21] |
| 22 | 1 | - | 1 | 1 | 1 | 1 | 1 | 1 | 1 | 1 | 1 | 1 | 1 | - | 12 | Good | [22] |
| 23 | 1 | 1 | - | 1 | 1 | 1 | 1 | 1 | 1 | 1 | 1 | 1 | 1 | 1 | 13 | Good | [23] |
| 24 | 1 | - | 1 | 1 | 1 | - | 1 | - | 1 | 1 | 1 | 1 | - | - | 9 | Fair | [24] |
| 25 | 1 | 0 | 0 | 1 | 1 | 1 | 0 | 1 | 1 | 1 | 1 | 1 | 0 | 0 | 9 | Fair | [25] |
| 26 | 1 | 0 | 1 | 1 | 1 | 1 | 0 | 1 | 1 | 1 | 1 | 1 | 0 | 0 | 10 | Good | [26] |
| 27 | 1 | 0 | 0 | 0 | 1 | 1 | 1 | 1 | 1 | 1 | 1 | 1 | 1 | 1 | 11 | Good | [27] |
| 28 | 1 | 0 | 1 | 1 | 1 | 1 | 0 | 1 | 1 | 1 | 1 | 1 | 0 | 0 | 10 | Good | [28] |
| 29 | 1 | 1 | 1 | 0 | 1 | 1 | 0 | 1 | 1 | 1 | 1 | 1 | 1 | 1 | 12 | Good | [29] |
| 30 | 1 | - | 1 | 1 | 1 | 1 | 1 | 1 | 1 | 1 | 1 | 1 | 1 | 1 | 13 | Good | [30] |
| 31 | 1 | 1 | 0 | 0 | 1 | 1 | 0 | 1 | 1 | 1 | 1 | 1 | 0 | 0 | 9 | Fair | [31] |
| 32 | 1 | 0 | 1 | 0 | 1 | 1 | 0 | 1 | 1 | 0 | 1 | 1 | 1 | 1 | 10 | Good | [32] |
| 33 | 1 | 1 | 1 | 0 | 1 | 1 | 1 | 1 | 1 | 1 | 1 | 1 | 0 | 1 | 12 | Good | [33] |
| 34 | 1 | 1 | 1 | 0 | 1 | 1 | 1 | 1 | 1 | 1 | 1 | 1 | 1 | 1 | 13 | Good | [34] |
| 35 | 1 | 0 | 1 | 1 | 1 | 1 | 0 | 0 | 1 | 1 | 1 | 1 | 1 | 1 | 11 | Good | [35] |
| 36 | 1 | 0 | 0 | 1 | 1 | 1 | 0 | 1 | 1 | 1 | 1 | 1 | 1 | 1 | 11 | Good | [36] |
| 37 | 1 | 0 | 0 | 0 | 1 | 1 | 1 | 1 | 1 | 1 | 1 | 1 | 1 | 1 | 11 | Good | [37] |
| 38 | 1 | 0 | 1 | 1 | 1 | 0 | 1 | 1 | 1 | 1 | 1 | 0 | 1 | 1 | 11 | Good | [38] |
| 39 | 1 | - | 1 | 1 | 1 | 1 | 1 | - | 1 | 1 | 1 | 1 | - | - | 10 | Good | [39] |
| 40 | 1 | 1 | 1 | 1 | 1 | 1 | 1 | 1 | 1 | 0 | 1 | 1 | 1 | 1 | 13 | Good | [40] |
| 41 | 1 | 0 | 0 | 0 | 1 | 1 | 0 | 1 | 1 | 1 | 1 | 1 | 0 | 0 | 8 | Fair | [41] |
| 42 | 1 | 0 | 1 | 1 | 1 | 1 | 1 | 0 | 1 | 1 | 1 | 1 | 1 | 0 | 11 | Good | [42] |
| 43 | 1 | - | 1 | 1 | 1 | 1 | 1 | 1 | 1 | 1 | 1 | 1 | 1 | 1 | 13 | Good | [43] |
| 44 | 1 | 1 | 1 | 1 | 1 | 1 | 1 | 1 | 1 | 1 | 1 | 1 | 1 | 1 | 14 | Good | [44] |
| 45 | 1 | 1 | 1 | 1 | 1 | 1 | 0 | 1 | 1 | 1 | 1 | 1 | 1 | 1 | 13 | Good | [45] |
| 46 | 1 | 0 | 1 | 1 | 0 | 1 | 0 | 1 | 1 | 1 | 1 | 1 | 0 | 0 | 9 | Fair | [46] |
| 47 | 1 | - | 1 | 1 | 1 | 1 | 1 | - | 1 | 1 | 1 | 1 | 1 | 1 | 12 | Good | [47] |
| 48 | 1 | 1 | 1 | 1 | 1 | 1 | 1 | 1 | 1 | 1 | 1 | 1 | 1 | 1 | 14 | Good | [48] |
| 49 | 1 | 0 | 1 | 1 | 1 | 1 | 0 | 1 | 1 | 1 | 1 | 1 | 0 | 0 | 10 | Good | [49] |
| 50 | 1 | 1 | 1 | 1 | 1 | 1 | 0 | 1 | 1 | 1 | 1 | 1 | 1 | 1 | 13 | Good | [50] |
| 51 | 1 | 1 | 0 | 1 | 1 | 1 | 0 | 1 | 1 | 1 | 1 | 1 | 1 | 0 | 11 | Good | [51] |
| 52 | 1 | 0 | 0 | 1 | 1 | 1 | 1 | 1 | 1 | 1 | 1 | 1 | 0 | 0 | 10 | Good | [52] |
| 53 | 1 | 0 | 0 | 1 | 1 | 1 | 1 | 1 | 1 | 1 | 1 | 1 | 1 | 1 | 12 | Good | [53] |
| 54 | 1 | 1 | 0 | 1 | 0 | 1 | 0 | 0 | 1 | 1 | 1 | 1 | 0 | 1 | 9 | Fair | [54] |
| 55 | 1 | 1 | 1 | - | 1 | 1 | 1 | 1 | 1 | 1 | 1 | 1 | 1 | 1 | 13 | Good | [55] |
| 56 | 1 | 0 | 1 | 0 | 1 | 1 | 1 | 1 | 1 | 0 | 1 | 0 | 1 | 0 | 9 | Fair | [56] |
| 57 | 1 | 0 | 1 | 1 | 1 | 1 | 1 | 1 | 1 | 1 | 1 | 1 | 1 | 1 | 13 | Good | [57] |
| 58 | 1 | 1 | 0 | 1 | 1 | 1 | 0 | 1 | 1 | 1 | 1 | 1 | 0 | 0 | 10 | Good | [58] |
| 59 | 1 | 0 | 0 | 1 | 1 | 1 | 1 | 1 | 1 | 1 | 1 | 1 | 1 | 1 | 12 | Good | [59] |
| 60 | 1 | 0 | 0 | 1 | 1 | 1 | 0 | 1 | 1 | 1 | 1 | 1 | 0 | 0 | 9 | Fair | [60] |
| 61 | 1 | 1 | - | 1 | 1 | 1 | 1 | 1 | 1 | 1 | 1 | 1 | 1 | 1 | 13 | Good | [61] |
| 62 | 1 | 1 | 1 | 1 | 1 | 1 | 1 | 1 | 1 | 1 | 1 | 1 | 1 | 1 | 14 | Good | [62] |
| 63 | 1 | 0 | 1 | 0 | 1 | 1 | 1 | 1 | 1 | 1 | 1 | 1 | 1 | 1 | 12 | Good | [63] |
| 64 | 1 | 0 | 1 | 1 | 1 | 1 | 1 | 1 | 1 | 1 | 1 | 1 | 1 | 0 | 12 | Good | [64] |
| 65 | 1 | 0 | 0 | 1 | 1 | 1 | 0 | 1 | 1 | 1 | 1 | 0 | 0 | 0 | 8 | Fair | [65] |
| 66 | 1 | - | 1 | 1 | 1 | 1 | 1 | 1 | 1 | 1 | 1 | 1 | 1 | 1 | 13 | Good | [66] |
| 67 | 1 | 1 | 1 | 1 | 1 | 1 | 0 | 1 | 1 | 1 | 1 | 1 | 1 | 0 | 12 | Good | [67] |
| 68 | 1 | 1 | 1 | 1 | 1 | 1 | 1 | 1 | 1 | 1 | 1 | 1 | 0 | 1 | 13 | Good | [68] |
| 69 | 1 | 1 | 1 | - | 1 | 1 | 1 | 1 | 1 | 1 | 1 | 1 | - | - | 11 | Good | [69] |
| 70 | 1 | 1 | 1 | 1 | 1 | 1 | 1 | 1 | 1 | 0 | 0 | 1 | 0 | 0 | 10 | Good | [70] |
| 71 | 1 | 0 | 1 | 1 | 1 | 1 | 0 | 1 | 1 | 1 | 1 | 0 | 0 | 0 | 9 | Fair | [71] |
| 72 | 1 | 0 | 1 | 0 | 1 | 1 | 1 | 1 | 1 | 1 | 1 | 1 | 1 | 0 | 11 | Good | [72] |
| 73 | 1 | 0 | 0 | 1 | 1 | 1 | 0 | 1 | 1 | 1 | 1 | 1 | 0 | 1 | 10 | Good | [73] |
| 74 | 1 | 1 | 0 | 0 | 1 | 1 | 1 | 1 | 1 | 1 | 1 | 1 | 1 | 0 | 11 | Good | [74] |
| 75 | 1 | 1 | 1 | 1 | 1 | 1 | 1 | 1 | 1 | 1 | 1 | 1 | 1 | 1 | 14 | Good | [75] |
| 76 | 1 | 1 | 1 | 1 | 1 | 1 | 1 | 1 | 1 | 1 | 1 | 1 | 1 | 1 | 14 | Good | [76] |
| 77 | 1 | 0 | 1 | 1 | 1 | 1 | 0 | 1 | 1 | 1 | 1 | 1 | 1 | 1 | 12 | Good | [77] |
| 78 | 1 | - | 1 | 1 | 1 | 1 | 1 | 1 | 1 | 1 | 1 | 1 | 1 | 1 | 13 | Good | [78] |
| 79 | 1 | 1 | 0 | 1 | 1 | 1 | 1 | 1 | 1 | 1 | 1 | 1 | 1 | 1 | 13 | Good | [79] |
| 80 | 1 | 1 | 0 | 1 | 1 | 1 | 0 | 1 | 1 | 1 | 1 | 1 | 0 | 0 | 10 | Good | [80] |
| 81 | 1 | 1 | 1 | 1 | 1 | 1 | 0 | 1 | 1 | 1 | 1 | 0 | 1 | 0 | 11 | Good | [81] |
| 82 | 1 | 0 | 0 | 1 | 1 | 1 | 0 | 1 | 1 | 1 | 1 | 1 | 1 | 1 | 11 | Good | [82] |
| 83 | 1 | 0 | 0 | 1 | 1 | 1 | 0 | 1 | 1 | 1 | 1 | 1 | 1 | 0 | 10 | Good | [83] |
| 84 | 1 | 1 | 0 | 0 | 1 | 1 | 1 | 1 | 1 | 1 | 1 | 1 | 0 | 0 | 10 | Good | [84] |
| 85 | 1 | 1 | 1 | 1 | 1 | 0 | 0 | 0 | 1 | 1 | 1 | 1 | 1 | 1 | 11 | Good | [85] |
| 86 | 1 | 1 | 1 | - | 1 | 1 | 1 | 1 | 1 | 1 | 1 | 1 | 1 | 1 | 13 | Good | [86] |
| 87 | 1 | 1 | 1 | 0 | 1 | 1 | 0 | 1 | 1 | 1 | 1 | 1 | 1 | 0 | 11 | Good | [87] |
| 88 | 1 | 1 | 1 | 1 | 1 | 1 | 1 | 1 | 1 | 1 | 1 | 1 | 0 | 0 | 12 | Good | [88] |
| 89 | 1 | 1 | 1 | 1 | 1 | 1 | 0 | 1 | 1 | 1 | 1 | 1 | 1 | 1 | 13 | Good | [89] |
| 90 | 1 | 1 | 1 | 0 | 1 | 1 | 1 | 1 | 1 | 1 | 1 | 1 | 1 | 1 | 13 | Good | [90] |
| 91 | 1 | 0 | 1 | 0 | 1 | 1 | 0 | 1 | 1 | 1 | 1 | 1 | 1 | 1 | 11 | Good | [91] |

^a)^ See Table 1 for the meaning of the code.

**Table S4.** Metals analysed in the reviewed articles

|  | **Frequency** | **Below Limit** | **Above Limit** |
| --- | --- | --- | --- |
|  | **n ^a)^ (%)** | **n ^b)^ (%)** | **n ^c)^ (%)** |
| **Essential Macro (n=6)** |  |  |  |
| Ca | 4 (66.7) | - | - |
| K | 3 (50.0) | 1 (100.0) | 0 (0.0) |
| Mg | 4 (66.7) | 1 (100.0) | 0 (0.0) |
| **Essential Micro (n=37)** |  |  |  |
| B | 1 (2.7) | - | - |
| Co | 10 (27.0) | 83 (100.0) | 0 (0.0) |
| Cr | 25 (67.6) | 353 (100.0) | 0 (0.0) |
| Cu | 29 (78.4) | 416 (99.3) | 3 (0.7) |
| Fe | 24 (64.9) | 333 (91.5) | 31 (8.5) |
| Mn | 26 (70.3) | 355 (95.9) | 15 (4.1) |
| Mo | 3 (8.1) | 45 (100.0) | 0 (0.0) |
| Na | 3 (8.1) | - | - |
| Ni | 22 (59.5) | 310 (98.7) | 4 (1.3) |
| Se | 4 (10.8) | 27 (100.0) | 0 (0.0) |
| Si | 1 (2.7) | - | - |
| Zn | 30 (81.1) | 397 (96.1) | 16 (3.9) |
| **Non-Essential (n=49)** |  |  |  |
| Ag | 1 (2.0) | 26 (100.0) | 0 (0.0) |
| Al | 7 (14.3) | 0 (0.0) | 1 (100.0) |
| As | 24 (49.0) | 228 (88.7) | 29 (11.3) |
| Ba | 4 (8.2) | 27 (93.1) | 2 (6.9) |
| Bi | 2 (4.1) | - | - |
| Cd | 38 (77.6) | 392 (81.7) | 88 (18.3) |
| Hg | 19 (38.8) | 201 (89.3) | 24 (10.7) |
| Pb | 43 (87.8) | 278 (42.4) | 377 (57.6) |
| Rb | 2 (4.1) | - | - |
| Sn | 1 (2.0) | 26 (100.0) | 0 (0.0) |
| Sr | 3 (6.1) | - | - |
| U | 2 (4.1) | - | - |
| V | 3 (6.1) | 38 (97.4) | 1 (2.6) |

^a)^ Number of peer-reviewed articles that measured the specific contaminant

^b)^ Number of data observations for each specific contaminant below limit

^c)^ Number of data observations for each specific contaminant above limit

**Table S5.** Microbial contaminants analysed in the reviewed articles

|  | **Frequency** | **Below Limit** | **Above Limit** |
| --- | --- | --- | --- |
|  | **n ^a)^ (%)** | **n ^b)^ (%)** | **n ^c)^ (%)** |
| **Bacteria (n=19)** |  |  |  |
| *Acinetobacter baumannii* | 1 (5.3) | 1 (100.0) | 0 (0.0) |
| *Acinetobacter calcoaceticus* | 1 (5.3) | 0 (0.0) | 1 (100.0) |
| *Acinetobacter lwoffii* | 1 (5.3) | 1 (100.0) | 0 (0.0) |
| *Acinetobacter species* | 1 (5.3) | 0 (0.0) | 3 (100.0) |
| *Aerobic bacteria* | 4 (21.1) | 49 (71.0) | 20 (29.0) |
| *Bacillus amyloliquefaciens* | 1 (5.3) | 1 (100.0) | 0 (0.0) |
| *Bacillus cereus* | 1 (5.3) | 0 (0.0) | 2 (100.0) |
| *Bacillus lentus* | 1 (5.3) | 1 (100.0) | 0 (0.0) |
| *Bacillus megaterium* | 2 (10.5) | 2 (28.6) | 5 (71.4) |
| *Bacillus polymyxa* | 1 (5.3) | 0 (0.0) | 7 (100.0) |
| *Bacillus pumilus* | 1 (5.3) | 0 (0.0) | 6 (100.0) |
| *Bacillus species* | 5 (26.3) | 57 (57.0) | 43 (43.0) |
| *Bacillus subtilis* | 2 (10.5) | 1 (7.1) | 13 (92.9) |
| *Bacillus vallismortis* | 1 (5.3) | 1 (100.0) | 0 (0.0) |
| *Citrobacter diversus* | 1 (5.3) | 0 (0.0) | 3 (100.0) |
| *Citrobacter intermidius* | 1 (5.3) | 0 (0.0) | 1 (100.0) |
| *Citrobacter species* | 1 (5.3) | 0 (0.0) | 4 (100.0) |
| *Clostridium species* | 1 (5.3) | 0 (0.0) | 8 (100.0) |
| *Coliforms* | 3 (15.8) | 22 (91.7) | 2 (8.3) |
| *Corynebacterium pseudodiphtheriticum* | 1 (5.3) | 0 (0.0) | 1 (100.0) |
| *Corynebacterium xerosis* | 1 (5.3) | 0 (0.0) | 1 (100.0) |
| *Enterobacter aerogenes* | 1 (5.3) | 0 (0.0) | 2 (100.0) |
| *Enterobacter cloacae* | 2 (10.5) | 1 (8.3) | 11 (91.7) |
| *Enterobacter species* | 1 (5.3) | 0 (0.0) | 1 (100.0) |
| *Enterobacteria species* | 1 (5.3) | 2 (16.7) | 10 (83.3) |
| *Enterococcus species* | 2 (10.5) | 0 (0.0) | 9 (100.0) |
| *Escherichia coli* | 10 (52.6) | 33 (42.3) | 45 (57.7) |
| *Klebsiella oxytoca* | 1 (5.3) | 1 (100.0) | 0 (0.0) |
| *Klebsiella ozaenae* | 1 (5.3) | 0 (0.0) | 3 (100.0) |
| *Klebsiella pneumoniae* | 2 (10.5) | 1 (6.3) | 15 (93.8) |
| *Lactobacillus casei* | 1 (5.3) | 0 (0.0) | 1 (100.0) |
| *Leclercia adecarboxylata* | 1 (5.3) | 1 (100.0) | 0 (0.0) |
| *Listeria grayi* | 1 (5.3) | 0 (0.0) | 2 (100.0) |
| *Listeria monocytogenes* | 1 (5.3) | 0 (0.0) | 2 (100.0) |
| *Listeria murrayi* | 1 (5.3) | 0 (0.0) | 3 (100.0) |
| *Micrococcus luteus* | 1 (5.3) | 0 (0.0) | 4 (100.0) |
| Not indicated | 3 (15.8) | 5 (100.0) | 0 (0.0) |
| *Pantoea species* | 1 (5.3) | 8 (100.0) | 0 (0.0) |
| *Proteus vulgaris* | 1 (5.3) | 0 (0.0) | 2 (100.0) |
| *Providencia species* | 1 (5.3) | 0 (0.0) | 2 (100.0) |
| *Providencia stuartii* | 1 (5.3) | 0 (0.0) | 4 (100.0) |
| *Pseudomonas aeruginosa* | 4 (21.1) | 5 (27.8) | 13 (72.2) |
| *Pseudomonas cetrimide* | 1 (5.3) | 4 (100.0) | 0 (0.0) |
| *Pseudomonas oryzihaitans* | 1 (5.3) | 2 (100.0) | 0 (0.0) |
| *Pseudomonas species* | 1 (5.3) | 5 (100.0) | 0 (0.0) |
| *Salmonella species* | 8 (42.1) | 29 (48.3) | 31 (51.7) |
| *Serratia marcescens* | 1 (5.3) | 0 (0.0) | 1 (100.0) |
| *Serratia species* | 1 (5.3) | 1 (20.0) | 4 (80.0) |
| *Shigella dysenteriae* | 1 (5.3) | 0 (0.0) | 13 (100.0) |
| *Shigella species* | 2 (10.5) | 4 (66.7) | 2 (33.3) |
| *Sphingomonas paucimobilis* | 1 (5.3) | 1 (100.0) | 0 (0.0) |
| *Staphylococcus aureus* | 5 (26.3) | 12 (31.6) | 26 (68.4) |
| *Staphylococcus epidermidis* | 3 (15.8) | 0 (0.0) | 10 (100.0) |
| *Staphylococcus saprophyticus* | 2 (10.5) | 1 (33.3) | 2 (66.7) |
| *Staphylococcus species* | 1 (5.3) | 2 (16.7) | 10 (83.3) |
| *Streptococcus faecalis* | 2 (10.5) | 10 (90.9) | 1 (9.1) |
| *Streptococcus mitis* | 1 (5.3) | 1 (100.0) | 0 (0.0) |
| *Streptococcus pyogenes* | 1 (5.3) | 0 (0.0) | 4 (100.0) |
| *Streptococcus species* | 1 (5.3) | 2 (16.7) | 10 (83.3) |
| **Fungi (n=13)** |  |  |  |
| *Actinomadura madurae* | 1 (7.7) | 0 (0.0) | 1 (100.0) |
| *Alternaria species* | 1 (7.7) | 3 (100.0) | 0 (0.0) |
| *Aspergillus flavus* | 3 (23.1) | 15 (71.4) | 6 (28.6) |
| *Aspergillus fumigatus* | 2 (15.4) | 5 (71.4) | 2 (28.6) |
| *Aspergillus nidulans* | 1 (7.7) | 0 (0.0) | 2 (100.0) |
| *Aspergillus niger* | 3 (23.1) | 11 (64.7) | 6 (35.3) |
| *Aspergillus oryzae* | 1 (7.7) | 0 (0.0) | 1 (100.0) |
| *Aspergillus species* | 2 (15.4) | 52 (94.5) | 3 (5.5) |
| *Candida albicans* | 2 (15.4) | 1 (11.1) | 8 (88.9) |
| *Candida pseudotropicalis* | 1 (7.7) | 0 (0.0) | 1 (100.0) |
| *Candida species* | 1 (7.7) | 10 (100.0) | 0 (0.0) |
| *Candida tropicalis* | 1 (7.7) | 0 (0.0) | 1 (100.0) |
| *Cladosporium species* | 2 (15.4) | 15 (100.0) | 0 (0.0) |
| *Cryptococcus neoformans* | 1 (7.7) | 0 (0.0) | 3 (100.0) |
| *Curvularia species* | 1 (7.7) | 5 (100.0) | 0 (0.0) |
| *Fungi species* | 3 (23.1) | 6 (75.0) | 2 (25.0) |
| *Fusarium species* | 1 (7.7) | 4 (100.0) | 0 (0.0) |
| *Geotricum species* | 1 (7.7) | 10 (100.0) | 0 (0.0) |
| *Hansenula anomala* | 1 (7.7) | 0 (0.0) | 4 (100.0) |
| *Madurella mycetomatis* | 1 (7.7) | 0 (0.0) | 1 (100.0) |
| Moulds | 5 (38.5) | 5 (17.9) | 23 (82.1) |
| *Mucor species* | 3 (23.1) | 20 (90.9) | 2 (9.1) |
| Not indicated | 3 (23.1) | 34 (85.0) | 6 (15.0) |
| *Penicillium species* | 3 (23.1) | 28 (73.7) | 10 (26.3) |
| *Rhizopus species* | 2 (15.4) | 6 (100.0) | 0 (0.0) |
| *Rhodotorula glutinis* | 1 (7.7) | 0 (0.0) | 2 (100.0) |
| *Saccharomyces cerevisiae* | 1 (7.7) | 0 (0.0) | 2 (100.0) |
| *Torulopsis candida* | 1 (7.7) | 0 (0.0) | 6 (100.0) |
| *Torulopsis glabrata* | 1 (7.7) | 0 (0.0) | 6 (100.0) |
| *Trichoderma harzianum* | 1 (7.7) | 0 (0.0) | 2 (100.0) |
| *Trichosporon cutaneum* | 1 (7.7) | 0 (0.0) | 2 (100.0) |
| **Not indicated ^d)^ (n=5)** | - | 60 (77.9) | 17 (22.1) |

^a)^ Number of peer-reviewed articles that measured the specific contaminant

^b)^ Number of data observations for each specific contaminant below limit

^c)^ Number of data observations for each specific contaminant above limit

^d)^ Number of data observations for which the specific type of microbial contaminant was not indicated

**Table S6.** Mycotoxins analysed in the reviewed articles

|  | **Frequency** | **Below Limit** | **Above Limit** |
| --- | --- | --- | --- |
|  | **n ^a)^ (%)** | **n ^b)^ (%)** | **n ^c)^ (%)** |
| **Aflatoxin (n=16)** |  |  |  |
| B1 | 14 (28.6) | 136 (72.3) | 52 (27.7) |
| B2 | 11 (22.5) | 30 (100.0) | 0 (0.0) |
| B3 | 1 (2.0) | - | - |
| G1 | 10 (20.4) | 38 (100.0) | 0 (0.0) |
| G1 + G2 | 1 (2.0) | 6 (100.0) | 0 (0.0) |
| G2 | 7 (14.3) | 30 (100.0) | 0 (0.0) |
| G3 | 1 (2.0) | - | - |
| Total aflatoxin | 4 (8.2) | 36 (81.8) | 8 (18.2) |
| **Fumonisins (n=4)** |  |  |  |
| B1 | 3 (60.0) | 2 (6.1) | 31 (93.9) |
| B2 | 1 (20.0) | 0 (0.0) | 2 (100.0) |
| Fumonisins | 1 (20.0) | 5 (100.0) | 0 (0.0) |
| **Ochratoxin (n=5)** |  |  |  |
| A | 5 (100.0) | 20 (58.8) | 14 (41.2) |
| **Patulin (n=2)** |  |  |  |
| Patulin | 2 (100.0) | 5 (83.3) | 1 (16.7) |
| **Trichothecenes (n=2)** |  |  |  |
| Diacetoxyscirpenol | 1 (100.0) | - | - |
| Deoxynivalenol | 1 (100.0) | 2 (100.0) | 0 (0.0) |
| **Zearalenone (n=2)** |  |  |  |
| Zearalenone | 2 (100.0) | 4 (80.0) | 1 (20.0) |

^a)^ Number of peer-reviewed articles that measured the specific contaminant

^b)^ Number of data observations for each specific contaminant below limit

^c)^ Number of data observations for each specific contaminant above limit

**Table S7.** Pesticides analysed in the reviewed articles

|  | **Frequency** | **Below Limit** | **Above Limit** |
| --- | --- | --- | --- |
|  | **n ^a)^ (%)** | **n ^b)^ (%)** | **n ^c)^ (%)** |
| **Organochlorine (n=5)** |  |  |  |
| 2,4-D | 1 (20.0) | 0 (0.0) | 1 (100.0) |
| Aldrin | 1 (20.0) | 30 (100.0) | 0 (0.0) |
| Chlordane cis | 1 (20.0) | 30 (100.0) | 0 (0.0) |
| DDT | 2 (40.0) | 2 (9.5) | 19 (90.5) |
| Dieldrin | 1 (20.0) | 30 (100.0) | 0 (0.0) |
| Endosulfan alpha | 2 (40.0) | 39 (100.0) | 0 (0.0) |
| Endosulfan beta | 1 (20.0) | 30 (100.0) | 0 (0.0) |
| Endosulfan sulphate | 1 (20.0) | 30 (100.0) | 0 (0.0) |
| Endrin | 1 (20.0) | 30 (100.0) | 0 (0.0) |
| HCH | 1 (20.0) | 0 (0.0) | 20 (100.0) |
| HCH beta | 1 (20.0) | 30 (100.0) | 0 (0.0) |
| HCH gamma | 1 (20.0) | 30 (100.0) | 0 (0.0) |
| Heptachlor | 1 (20.0) | 30 (100.0) | 0 (0.0) |
| Methoxychlor | 1 (20.0) | 30 (100.0) | 0 (0.0) |
| Total HCH | 1 (20.0) | 3 (33.3) | 6 (66.7) |
| o, p’-DDT | 1 (20.0) | 9 (100.0) | 0 (0.0) |
| p, p’-DDD | 2 (40.0) | 39 (100.0) | 0 (0.0) |
| p, p’-DDE | 2 (40.0) | 39 (100.0) | 0 (0.0) |
| p, p’-DDT | 2 (40.0) | 39 (100.0) | 0 (0.0) |
| α-BHC | 1 (20.0) | 3 (100.0) | 0 (0.0) |
| α-HCH | 1 (20.0) | 9 (100.0) | 0 (0.0) |
| β-BHC | 1 (20.0) | 3 (100.0) | 0 (0.0) |
| β-HCH | 2 (40.0) | 9 (100.0) | 0 (0.0) |
| γ-HCH | 1 (20.0) | 9 (100.0) | 0 (0.0) |
| γ-BHC | 2 (40.0) | 4 (100.0) | 0 (0.0) |
| δ-BHC | 1 (20.0) | 3 (100.0) | 0 (0.0) |
| δ-HCH | 1 (20.0) | 9 (100.0) | 0 (0.0) |
| **Organophosphate (n=2)** |  |  |  |
| Chlorfenvinphos | 1 (33.3) | 30 (100.0) | 0 (0.0) |
| Chlorpyrifos | 1 (33.3) | 30 (100.0) | 0 (0.0) |
| Diazinon | 1 (33.3) | 30 (100.0) | 0 (0.0) |
| Dimethoate | 1 (33.3) | 30 (100.0) | 0 (0.0) |
| Ethoprophos | 1 (33.3) | 30 (100.0) | 0 (0.0) |
| Fenitrothion | 1 (33.3) | 30 (100.0) | 0 (0.0) |
| Fonofos | 1 (33.3) | 30 (100.0) | 0 (0.0) |
| Malathion | 2 (66.7) | 31 (100.0) | 0 (0.0) |
| Methamidophos | 1 (33.3) | 30 (100.0) | 0 (0.0) |
| Parathion ethyl | 1 (33.3) | 30 (100.0) | 0 (0.0) |
| Pirimiphos methyl | 1 (33.3) | 30 (100.0) | 0 (0.0) |
| Profenofos | 1 (33.3) | 30 (100.0) | 0 (0.0) |
| **Pyrethroid (n=1)** |  |  |  |
| Bifenthrin | 1 (100.0) | 30 (100.0) | 0 (0.0) |
| Cyfluthrin I | 1 (100.0) | 30 (100.0) | 0 (0.0) |
| Cyfluthrin II | 1 (100.0) | 30 (100.0) | 0 (0.0) |
| Cyfluthrin III | 1 (100.0) | 30 (100.0) | 0 (0.0) |
| Cyfluthrin IV | 1 (100.0) | 30 (100.0) | 0 (0.0) |
| Cyhalothrin lambda | 1 (100.0) | 30 (100.0) | 0 (0.0) |
| Cypermethrin I | 1 (100.0) | 30 (100.0) | 0 (0.0) |
| Cypermethrin II | 1 (100.0) | 30 (100.0) | 0 (0.0) |
| Cypermethrin III | 1 (100.0) | 30 (100.0) | 0 (0.0) |
| Cypermethrin IV | 1 (100.0) | 30 (100.0) | 0 (0.0) |
| Deltamethrin | 1 (100.0) | 30 (100.0) | 0 (0.0) |
| Fenpropathrin | 1 (100.0) | 30 (100.0) | 0 (0.0) |
| Fenvalerate I | 1 (100.0) | 30 (100.0) | 0 (0.0) |
| Fenvalerate II | 1 (100.0) | 30 (100.0) | 0 (0.0) |
| Permethrin cis | 1 (100.0) | 30 (100.0) | 0 (0.0) |
| Permethrin trans | 1 (100.0) | 30 (100.0) | 0 (0.0) |

^a)^ Number of peer-reviewed articles that measured the specific contaminant

^b)^ Number of data observations for each specific contaminant below limit

^c)^ Number of data observations for each specific contaminant above limit

**Table S8.** Residual solvents analysed in the reviewed articles

|  | **Frequency** | **Below Limit** | **Above Limit** |
| --- | --- | --- | --- |
|  | **n ^a)^ (%)** | **n ^b)^ (%)** | **n ^c)^ (%)** |
| **Class 1 (n=2)** |  |  |  |
| 1,1,1-Trichloroethane | 1 (50.0) | 7 (100.0) | 0 (0.0) |
| 1,1-Dichloroethene | 1 (50.0) | 7 (100.0) | 0 (0.0) |
| 1,2-Dichloroethane | 1 (50.0) | 7 (100.0) | 0 (0.0) |
| Benzene | 2 (100.0) | 9 (75.0) | 3 (25.0) |
| Carbon Tetrachloride | 2 (100.0) | 12 (100.0) | 0 (0.0) |
| **Class 2 (n=4)** |  |  |  |
| 1,2-Dimethoxyethane | 1 (25.0) | 7 (100.0) | 0 (0.0) |
| 1,4-Dioxane | 1 (25.0) | 7 (100.0) | 0 (0.0) |
| 2,3-Pentadione | 1 (25.0) | 3 (100.0) | 0 (0.0) |
| Acetonitrile | 3 (75.0) | 37 (100.0) | 0 (0.0) |
| Chlorobenzene | 1 (25.0) | 7 (100.0) | 0 (0.0) |
| Chloroform | 1 (25.0) | 6 (85.7) | 1 (14.3) |
| Cis-1,2-Dichloroethene | 1 (25.0) | 7 (100.0) | 0 (0.0) |
| Cumene | 1 (25.0) | 7 (100.0) | 0 (0.0) |
| Cyclohexane | 2 (50.0) | 12 (100.0) | 0 (0.0) |
| Dichloromethane | 1 (25.0) | 5 (100.0) | 0 (0.0) |
| Ethylbenzene | 1 (25.0) | 7 (100.0) | 0 (0.0) |
| Hexane | 3 (75.0) | 37 (100.0) | 0 (0.0) |
| Methanol | 3 (75.0) | 35 (100.0) | 0 (0.0) |
| Methylbutylketone | 1 (25.0) | 7 (100.0) | 0 (0.0) |
| Methylcyclohexane | 1 (25.0) | 7 (100.0) | 0 (0.0) |
| Methylene Chloride | 1 (25.0) | 7 (100.0) | 0 (0.0) |
| Nitromethane | 1 (25.0) | 7 (100.0) | 0 (0.0) |
| Pyridine | 1 (25.0) | 7 (100.0) | 0 (0.0) |
| Tetrahydrofuran | 2 (50.0) | 12 (100.0) | 0 (0.0) |
| Tetralin | 1 (25.0) | 7 (100.0) | 0 (0.0) |
| Toluene | 3 (75.0) | 37 (100.0) | 0 (0.0) |
| Trans-1,2-Dichloroethene | 1 (25.0) | 7 (100.0) | 0 (0.0) |
| Trichloroethylene | 1 (25.0) | 7 (100.0) | 0 (0.0) |
| Xylene | 1 (25.0) | 5 (100.0) | 0 (0.0) |
| m-Xylene & p-Xylene | 1 (25.0) | 7 (100.0) | 0 (0.0) |
| o-Xylene | 1 (25.0) | 7 (100.0) | 0 (0.0) |
| **Class 3 (n=4)** |  |  |  |
| 1-Butanol | 2 (50.0) | 28 (100.0) | 0 (0.0) |
| 1-Propanol | 1 (25.0) | 3 (100.0) | 0 (0.0) |
| 2-Butanol | 1 (25.0) | 3 (100.0) | 0 (0.0) |
| 2-Propanol | 1 (25.0) | 3 (100.0) | 0 (0.0) |
| Acetic acid | 1 (25.0) | 3 (100.0) | 0 (0.0) |
| Acetone | 3 (75.0) | 33 (100.0) | 0 (0.0) |
| Ethanol | 3 (75.0) | 33 (89.2) | 4 (10.8) |
| Ethyl acetate | 3 (75.0) | 33 (100.0) | 0 (0.0) |
| Ethyl ether | 1 (25.0) | 5 (100.0) | 0 (0.0) |
| Isoamyl alcohol | 1 (25.0) | - | - |
| Isobutanol | 1 (25.0) | 1 (100.0) | 0 (0.0) |
| n-Butyl acetate | 1 (25.0) | 3 (100.0) | 0 (0.0) |
| **Not Classified (n=2)** |  |  |  |
| 1-Hexanol | 1 (50.0) | 3 (100.0) | 0 (0.0) |
| Acetoin | 1 (50.0) | 2 (100.0) | 0 (0.0) |
| Diacetyl | 1 (50.0) | 3 (100.0) | 0 (0.0) |
| Furfural | 1 (50.0) | 3 (100.0) | 0 (0.0) |
| Isoamyl acetate | 1 (50.0) | 3 (100.0) | 0 (0.0) |
| Octane | 1 (50.0) | 5 (100.0) | 0 (0.0) |
| Styrene | 1 (50.0) | 3 (100.0) | 0 (0.0) |

^a)^ Number of peer-reviewed articles that measured the specific contaminant

^b)^ Number of data observations for each specific contaminant below limit

^c)^ Number of data observations for each specific contaminant above limit

**Table S9.** Analytical methods used for the analysis of the contaminants in the reviewed articles

|  | **Frequency** | **Percent** |
| --- | --- | --- |
| **Metal (n=51)** |  |  |
| AAS | 26 | 51.0 |
| GC | 1 | 2.0 |
| GC-MS; ICP-MS | 1 | 2.0 |
| ICP-MS | 10 | 19.6 |
| ICPS | 12 | 23.5 |
| ICPS; AFS | 1 | 2.0 |
| INAA | 1 | 2.0 |
| Voltammetry | 1 | 2.0 |
| **Microbial (n=25)** |  |  |
| Microbial culture | 25 | 100.0 |
| HPLC | 1 | 4.0 |
| **Mycotoxins (n=17)** |  |  |
| FAPS | 1 | 5.9 |
| HPLC | 10 | 58.8 |
| HPLC-MS | 2 | 11.8 |
| Immunoassay | 1 | 5.9 |
| TLC | 1 | 5.9 |
| TLC & HPLC | 1 | 5.9 |
| UPLC-MS | 1 | 5.9 |
| **Pesticide (n=5)** |  |  |
| GC | 4 | 80.0 |
| GC-MS | 1 | 20.0 |
| **Residual Solvent (n=4)** |  |  |
| GC | 4 | 100.0 |

AAS, Atomic absorption spectroscopy; FAPS, Fluorescent Aptasensor PicoGreen-Based Strategy; GC, Gas chromatography; GC-MS, Gas chromatography mass spectrometry; ICP-MS, Inductively coupled plasma mass spectrometry; ICPS, Inductively coupled plasma spectroscopy; HPLC, High performance liquid chromatography; AFS, Atomic fluorescence spectrometry; HPLC-MS, High performance liquid chromatography mass spectrometry; INAA, Instrumental neutron activation analysis; TLC, Thin-layer chromatography; UPLC-MS, Ultra performance liquid chromatography mass spectrometry.

**Supplementary figures**

**Figure S1.** Continental distribution of the reviewed articles

**Figure S2** Number of contaminants measured per reviewed article

**Figure S3.** Types of contaminants reported in the reviewed articles.

**References**

1. Baxter, R. and W.H. Holzapfel, *A Microbial Investigation of Selected Spices, Herbs, and Additives in South Africa.* Journal of Food Science, 1982. **47**(2): p. 570-574.

2. Steenkamp, V., M. von Arb, and M.J. Stewart, *Metal concentrations in plants and urine from patients treated with traditional remedies.* Forensic Sci Int, 2000. **114**(2): p. 89-95.

3. Bogusz, M.J., M. al Tufail, and H. Hassan, *How natural are 'natural herbal remedies'? A Saudi perspective.* Adverse Drug React Toxicol Rev, 2002. **21**(4): p. 219-29.

4. Liu, J., et al., *Determination of volatile residual solvents in traditional Chinese medicines by headspace solid-phase microextraction and cryogenic gas chromatography with flame ionization detection.* J AOAC Int, 2003. **86**(3): p. 461-6.

5. Tassaneeyakul, W., et al., *Contamination of aflatoxins in herbal medicinal products in Thailand.* Mycopathologia, 2004. **158**(2): p. 239-44.

6. Steenkamp, V., et al., *Uranium concentrations in South African herbal remedies.* Health Phys, 2005. **89**(6): p. 679-83.

7. Govender, S., et al., *Traditional herbal medicines: microbial contamination, consumer safety and the need for standards.* South African journal of science, 2006. **102**(5): p. 253-255.

8. Nnorom, I., O. Osibanjo, and C. Eleke, *Evaluation of human exposure to Lead and Cadmium from some local Nigerian Medicinal preparations.* Journal of Applied Sciences, 2006. **6**(14): p. 2907-2911.

9. Srivastava, S.K., et al., *Estimation of heavy metals in different berberis species and its market samples.* Environ Monit Assess, 2006. **116**(1-3): p. 315-20.

10. Steenkamp, V., E. Cukrowska, and M.J. Stewart, *Metal concentrations in South African traditional herbal remedies.* South African journal of science, 2006. **102**(5): p. 256-258.

11. Du, B. and H. Liu, *Determination of Organochlorine Pesticide Residues in Herbs by Capillary Electrophoresis.* Life Science Journal, 2007. **4**(1): p. 40-42.

12. Esimone, C., et al., *Susceptibility-resistance profile of micro-organisms isolated from herbal medicine products sold in Nigeria.* African Journal of Biotechnology, 2007. **6**(24).

13. Mishra, C., S. Sharma, and P. Kakkar, *A study to evaluate heavy metals and organochlorine pesticide residue in Zingiber officinale Rosc. collected from different ecological zones of India.* Bull Environ Contam Toxicol, 2007. **79**(1): p. 95-8.

14. Katerere, D.R., et al., *A preliminary survey of mycological and fumonisin and aflatoxin contamination of African traditional herbal medicines sold in South Africa.* Hum Exp Toxicol, 2008. **27**(11): p. 793-8.

15. Street, R.A., et al., *Variation in heavy metals and microelements in South African medicinal plants obtained from street markets.* Food Addit Contam Part A Chem Anal Control Expo Risk Assess, 2008. **25**(8): p. 953-60.

16. Cho, S.Y., et al., *Co-contamination of Aflatoxins with Ochratoxin A and Zearalenone in Thuja orientalis Semen.* Toxicol Res, 2009. **25**(3): p. 125-131.

17. Bhat, R., et al., *Determination of Mineral Composition and Heavy Metal Content of Some Nutraceutically Valued Plant Products.* Food Analytical Methods, 2010. **3**(3): p. 181-187.

18. Meena, A.K., et al., *Estimation of heavy metals in commonly used medicinal plants: a market basket survey.* Environ Monit Assess, 2010. **170**(1-4): p. 657-60.

19. Meos, A., et al., *Lead content in pot marigold (Calendula officinalis L.) inflorescences and leaves: impact of precipitations and vicinity of motorway.* Biol Trace Elem Res, 2011. **140**(2): p. 244-51.

20. Iqbal, S.Z., et al., *Comparing aflatoxin contamination in chilies from Punjab, Pakistan produced in summer and winter.* Mycotoxin Res, 2011. **27**(2): p. 75-80.

21. Pakade, Y.B., et al., *Metals in herbal drugs from Himalayan region.* Bull Environ Contam Toxicol, 2011. **86**(1): p. 133-6.

22. Rao, M.M., A. Kumarmeena, and Galib, *Detection of toxic heavy metals and pesticide residue in herbal plants which are commonly used in the herbal formulations.* Environ Monit Assess, 2011. **181**(1-4): p. 267-71.

23. Wu, J., et al., *Toxic metal contamination in Artemisia annua L. herbal preparations from different commercial sources in China.* Journal of Natural Medicines, 2011. **65**(3): p. 656-661.

24. Ahmad, M.I., et al., *Pharmacognostical studies and establishment of quality parameters of Cucmis melo L. CV. Namdhari.* Int J Pharm Pharm Sci, 2012. **4**: p. 324-9.

25. Bempah, C.K., et al., *Heavy metals contamination in herbal plants from some Ghanaian markets.* Journal of Microbiology, Biotechnology and Food Sciences, 2012. **2**(3): p. 886-896.

26. Ezekiel, C.N., et al., *Multi-microbial metabolites in fonio millet (acha) and sesame seeds in Plateau State, Nigeria.* European Food Research and Technology, 2012. **235**(2): p. 285-293.

27. Kaume, L., J.C. Foote, and E.E. Gbur, *Microbial contamination of herbs marketed to HIV-infected people in Nairobi (Kenya).* South African Journal of Science, 2012. **108**(9): p. 1-4.

28. Liu, L., et al., *Determination of aflatoxins in medicinal herbs by high-performance liquid chromatography-tandem mass spectrometry.* Phytochem Anal, 2012. **23**(5): p. 469-76.

29. Okem, A., et al., *Determination of total and bioavailable heavy and trace metals in South African commercial herbal concoctions using ICP-OES.* South African Journal of Botany, 2012. **82**: p. 75-82.

30. Alomary, A., et al., *Pb in medicinal plants from Jordan.* Environmental Chemistry Letters, 2013. **11**(1): p. 55-63.

31. Manwar, J., et al., *Gas chromatography method for the determination of non-ethanol volatile compounds in herbal formulation.* International Journal of Analytical and Bioanalytical Chemistry, 2013. **3**(1): p. 12-17.

32. Noor, R., et al., *Microbial contamination in herbal medicines available in Bangladesh.* Bangladesh Med Res Counc Bull, 2013. **39**(3): p. 124-9.

33. Ozdemir, B., et al., *How safe is the use of herbal weight-loss products sold over the internet?* Hum Exp Toxicol, 2013. **32**(1): p. 101-6.

34. Rasdi, F.L., N.K. Bakar, and S. Mohamad, *A Comparative Study of Selected Trace Element Content in Malay and Chinese Traditional Herbal Medicine (THM) Using an Inductively Coupled Plasma-Mass Spectrometer (ICP-MS).* Int J Mol Sci, 2013. **14**(2): p. 3078-93.

35. Shah, A., et al., *Comparative Study of Heavy Metals in Soil and Selected Medicinal Plants.* Journal of Chemistry, 2013. **2013**: p. 621265.

36. Ayaz, M., et al., *Heavy metals analysis, phytochemical, phytotoxic and anthelmintic investigations of crude methanolic extract, subsequent fractions and crude saponins from Polygonum hydropiper L.* BMC Complement Altern Med, 2014. **14**: p. 465.

37. Ezekwesili-Ofili, J., et al., *The bioload and aflatoxin content of herbal medicines from selected states in Nigeria.* African Journal of Traditional, Complementary and Alternative Medicines, 2014. **11**(3): p. 143-147.

38. Namdari, F., et al., *A survey on microbial quality of herbal distillates in Isfahan, central of Iran.* Studia Universitatis Vasile Goldis Arad, Seria Stiintele Vietii, 2014. **24**(4): p. 407-411.

39. Ullah, N., I. Ahmad, and S. Ayaz, *In vitro antimicrobial and antiprotozoal activities, phytochemical screening and heavy metals toxicity of different parts of Ballota nigra.* Biomed Res Int, 2014. **2014**: p. 321803.

40. Van Vuuren, S., et al., *Microbial contamination of traditional medicinal plants sold at the Faraday muthi market, Johannesburg, South Africa.* South African Journal of Botany, 2014. **94**: p. 95-100.

41. ALFaris, N.A., R.M. Al Ashban, and M. Al Ojayan, *Safety evaluation of local weight-gain formulas in the Saudi Arabian markets.* ScientificWorldJournal, 2015. **2015**: p. 136097.

42. Chen, A.J., et al., *Mycobiota and Mycotoxins in Traditional Medicinal Seeds from China.* Toxins (Basel), 2015. **7**(10): p. 3858-75.

43. Dghaim, R., et al., *Determination of Heavy Metals Concentration in Traditional Herbs Commonly Consumed in the United Arab Emirates.* Journal of Environmental and Public Health, 2015. **2015**: p. 973878.

44. Kanu, A., O.A. Igbeneghu, and L.O. Orafidiya, *The organoleptic and microbial quality of some herbal medicinal products marketed in Freetown, Sierra Leone.* African Journal of Traditional, Complementary and Alternative Medicines, 2015. **12**(4): p. 1-8.

45. Moore, F., et al., *Potential Health Risk of Herbal Distillates and Decoctions Consumption in Shiraz, Iran.* Biol Trace Elem Res, 2015. **167**(2): p. 326-37.

46. Muller, A.L.H., et al., *Microwave-assisted digestion using diluted acids for toxic element determination in medicinal plants by ICP-MS in compliance with United States pharmacopeia requirements.* Analytical Methods, 2015. **7**(12): p. 5218-5225.

47. Sadhu, A., et al., *Quantitative analysis of heavy metals in medicinal plants collected from environmentally diverse locations in India for use in a novel phytopharmaceutical product.* Environ Monit Assess, 2015. **187**(8): p. 542.

48. Zamir, R., et al., *Microbial and Heavy Metal Contaminant of Antidiabetic Herbal Preparations Formulated in Bangladesh.* Evid Based Complement Alternat Med, 2015. **2015**: p. 243593.

49. Aiko, V. and A. Mehta, *Prevalence of toxigenic fungi in common medicinal herbs and spices in India.* 3 Biotech, 2016. **6**(2): p. 159.

50. de Bruin, W., D. Otto, and L. Korsten, *Microbiological Status and Food Safety Compliance of Commercial Basil Production Systems.* J Food Prot, 2016. **79**(1): p. 43-50.

51. Mahlangeni, N.T., R. Moodley, and S.B. Jonnalagadda, *Heavy metal distribution in Laportea peduncularis and growth soil from the eastern parts of KwaZulu-Natal, South Africa.* Environ Monit Assess, 2016. **188**(2): p. 76.

52. Naz, N., et al., *Quantitative Scrutinization of Aflatoxins in Different Spices from Pakistan.* Int J Anal Chem, 2016. **2016**: p. 4907425.

53. Nkansah, M.A., et al., *Heavy metal contents of some medicinal herbs from Kumasi, Ghana.* Cogent Environmental Science, 2016. **2**(1): p. 1234660.

54. Seddigi, Z.S., et al., *Assessment of metal contents in spices and herbs from Saudi Arabia.* Toxicol Ind Health, 2016. **32**(2): p. 260-9.

55. Tosun, H., P.G. Ergönül, and E.F. Üçok, *Occurrence of aflatoxins (B1, B2, G1, G2) in herbal tea consumed in Turkey.* Journal für Verbraucherschutz und Lebensmittelsicherheit, 2016. **11**(3): p. 265-269.

56. Walther, C., et al., *Microbial contamination of traditional liquid herbal medicinal products marketed in Mwanza city: magnitude and risk factors.* Pan Afr Med J, 2016. **23**: p. 65.

57. Yesuf, A., et al., *Occurrence of Potential Bacterial Pathogens and Their Antimicrobial Susceptibility Patterns Isolated from Herbal Medicinal Products Sold in Different Markets of Gondar Town, Northwest Ethiopia.* Int J Bacteriol, 2016. **2016**: p. 1959418.

58. Benson, R.A., et al., *Application of k0-INAA for the determination of essential and toxic elements in medicinal plants from West Pokot County, Kenya.* Journal of Radioanalytical and Nuclear Chemistry, 2017. **314**(1): p. 23-29.

59. Das, A. and A. Das, *Heavy metals in common food items in Kolkata, India.* Euro-Mediterranean Journal for Environmental Integration, 2017. **3**(1): p. 1.

60. Gupta, D., P. Bala, and Y.P. Sharma, *Evaluation of Fungal Flora and Mycotoxin Contamination in Whole Dried Apricots (Prunus armeniaca L.) from J&K, India.* Proceedings of the National Academy of Sciences, India Section B: Biological Sciences, 2017. **87**(1): p. 81-87.

61. Mulaudzi, R.B., et al., *Antimicrobial activity, cytotoxicity evaluation and heavy metal content of five commonly used South African herbal mixtures.* South African Journal of Botany, 2017. **112**: p. 314-318.

62. Siriangkhawut, W., et al., *Quality assessment of trace Cd and Pb contaminants in Thai herbal medicines using ultrasound-assisted digestion prior to flame atomic absorption spectrometry.* Journal of Food and Drug Analysis, 2017. **25**(4): p. 960-967.

63. Aghaji, A.E., I.V. Ezeome, and E.R. Ezeome, *Evaluation of content and cost of traditional eye medication in a resource-poor country - Implications for eye care practice and policy.* Niger J Clin Pract, 2018. **21**(11): p. 1514-1519.

64. Akpo-Djenontin, D.O.O., et al., *Mold infestation and aflatoxins production in traditionally processed spices and aromatic herbs powder mostly used in West Africa.* Food Sci Nutr, 2018. **6**(3): p. 541-548.

65. Gounden, T., R. Moodley, and S.B. Jonnalagadda, *Elemental analysis and nutritional value of edible Trifolium (clover) species.* J Environ Sci Health B, 2018. **53**(8): p. 487-492.

66. Kumar, N., et al., *Profiling of heavy metal and pesticide residues in medicinal plants.* Environ Sci Pollut Res Int, 2018. **25**(29): p. 29505-29510.

67. Li, K., et al., *Multielements determination and metal transfer investigation in herb medicine Bupleuri Radix by inductively coupled plasma-mass spectrometry.* Food Sci Nutr, 2018. **6**(8): p. 2005-2014.

68. Ozyigit, II, et al., *Investigation of Heavy Metal Level and Mineral Nutrient Status in Widely Used Medicinal Plants' Leaves in Turkey: Insights into Health Implications.* Biol Trace Elem Res, 2018. **182**(2): p. 387-406.

69. Zhang, C., et al., *A Rapid Label-Free Fluorescent Aptasensor PicoGreen-Based Strategy for Aflatoxin B(1) Detection in Traditional Chinese Medicines.* Toxins (Basel), 2018. **10**(3).

70. Abualhasan, M., et al., *Evaluation of Heavy Metals and Microbiological Contamination of Selected Herbals from Palestine.* Open Life Sci, 2019. **14**: p. 448-453.

71. Gounden, T., R. Moodley, and S.B. Jonnalagadda, *Distribution and Assessment of Heavy Metals in Trifolium dubium (Little Hop Clover) and the Impact of Soil Quality.* Analytical Letters, 2019. **52**(7): p. 1165-1176.

72. Ideh, J.E. and A.T. Ogunkunle, *User frequency and microbial contaminants of traditional oral powdered herbal formulations in Ogbomoso, Nigeria.* Journal of Medicinal Plants for Economic Development, 2019. **3**(1): p. 1-9.

73. Kohzadi, S., et al., *Concentration, Source, and Potential Human Health Risk of Heavy Metals in the Commonly Consumed Medicinal Plants.* Biol Trace Elem Res, 2019. **187**(1): p. 41-50.

74. Maithani, M., et al., *Assessment of compliance level of ICH guidelines for organic volatile impurities in common ayurvedic hepatic formulations.* J Complement Integr Med, 2019. **16**(3).

75. Mngadi, S., R. Moodley, and S.B. Jonnalagadda, *Elemental composition and nutritional value of the edible fruits of Transvaal red milkwood (Mimusops zeyheri) and impact of soil quality.* Environ Monit Assess, 2019. **191**(3): p. 135.

76. Ngemenya, M.N., et al., *Microbial, phytochemical, toxicity analyses and antibacterial activity against multidrug resistant bacteria of some traditional remedies sold in Buea Southwest Cameroon.* BMC Complementary and Alternative Medicine, 2019. **19**(1): p. 150.

77. Ofusori, A.E., R. Moodley, and S.B. Jonnalagadda, *Elemental distribution in the edible leaves of Celosia trigyna from the western and northern regions of Nigeria.* J Environ Sci Health B, 2019. **54**(1): p. 61-69.

78. Olaniyi, M.B., I.O. Lawal, and S.O. Rufai, *Evaluation of heavy metals in some selected medicinal plants growing within the University of Ibadan Campus.* Journal of Medicinal Plants for Economic Development, 2019. **3**(1): p. 1-6.

79. Tadesse, A.W., et al., *Determination of Heavy Metal Concentrations and Their Potential Sources in Selected Plants: Xanthium strumarium L. (Asteraceae), Ficus exasperata Vahl (Moraceae), Persicaria attenuata (R.Br) Sojak (Polygonaceae), and Kanahia laniflora (Forssk.) R.Br. (Asclepiadaceae) from Awash River Basin, Ethiopia.* Biol Trace Elem Res, 2019. **191**(1): p. 231-242.

80. Abed, R., et al., *Investigation of fungi and mycotoxins contamination in some herbal slimming mixtures in Baquba city-Iraq.* Microbial Biosystems, 2020. **5**(1): p. 1-6.

81. Arienzo, A., et al., *Microbiological Quality of Ready-to-Eat Leafy Green Salads during Shelf-Life and Home-Refrigeration.* Foods, 2020. **9**(10).

82. de Sousa Lima, C.M., et al., *Microbial contamination in herbal medicines: a serious health hazard to elderly consumers.* BMC Complement Med Ther, 2020. **20**(1): p. 17.

83. Guragain, B., et al., *Correlative study of heavy metal content with biological importance of Solanum virginianum leaf extract.* Clinical Phytoscience, 2020. **6**(1): p. 81.

84. Ikeagwulonu, R.C., et al., *Mycotoxin contamination of herbal medications on sale in Ebonyi State, Nigeria.* International Journal of Biological and Chemical Sciences, 2020. **14**(2): p. 613-625.

85. Jayani, N.I.E., K. Kartini, and L.K. Putri, *Standardization of a Crude Drug Moringa oleifera Leaf from Africa, India and Local (Indonesian) which Cultivated in Bojonegoro Indonesia.* International Journal of Pharmaceutical Research, 2020. **12**(1).

86. Nath, A., D. Chakraborty, and S. Das, *Assessment of lead and cadmium in fifty-four Indian herbal medicine: tribal and marketed varieties.* Environ Sci Pollut Res Int, 2020. **27**(4): p. 4127-4136.

87. Turkson, B.K., et al., *Evaluation of the Microbial Load and Heavy Metal Content of Two Polyherbal Antimalarial Products on the Ghanaian Market.* Evid Based Complement Alternat Med, 2020. **2020**: p. 1014273.

88. Wikandari, R., et al., *Assessment of Microbiological Quality and Mycotoxin in Dried Chili by Morphological Identification, Molecular Detection, and Chromatography Analysis.* International Journal of Environmental Research and Public Health, 2020. **17**(6): p. 1847.

89. Odukoya, J.O., J.O. Odukoya, and D.T. Ndinteh, *Elemental measurements and health risk assessment of sub-Saharan African medicinal plants used for cardiovascular diseases' and related risk factors' treatment.* J Trace Elem Med Biol, 2021. **65**: p. 126725.

90. Opuni, K.F.M., et al., *Monitoring and risk assessment of pesticide residues in selected herbal medicinal products in Ghana.* Environ Monit Assess, 2021. **193**(8): p. 470.

91. Opuni, K.F.M., et al., *Monitoring of residual solvent contamination in herbal medicinal products in Ghana: A pilot study.* Scientific African, 2021. **13**: p. e00825.
